# Metal Oxide and Hydroxide–Based Aqueous Supercapacitors: From Charge Storage Mechanisms and Functional Electrode Engineering to Need‐Tailored Devices

**DOI:** 10.1002/advs.201801797

**Published:** 2019-02-13

**Authors:** Tuyen Nguyen, Maria de Fátima Montemor

**Affiliations:** ^1^ Centro de Química Estrutural (CQE) Departamento de Engenharia Química (DEQ) Instituto Superior Técnico Universidade de Lisboa 1049‐001 Lisbon Portugal

**Keywords:** aqueous supercapacitors, metal hydroxides, metal oxides, storage mechanisms, supercapacitor electrodes

## Abstract

Energy storage devices that efficiently use energy, in particular renewable energy, are being actively pursued. Aqueous redox supercapacitors, which operate in high ionic conductivity and environmentally friendly aqueous electrolytes, storing and releasing high amounts of charge with rapid response rate and long cycling life, are emerging as a solution for energy storage applications. At the core of these devices, electrode materials and their assembling into rational configurations are the main factors governing the charge storage properties of supercapacitors. Redox‐active metal compounds, particularly oxides and hydroxides that store charge via reversible valence change redox reactions with electrolyte ions, are prospective candidates to optimize the electrochemical performance of supercapacitors. To address this target, collaborative investigations, addressing different streams, from fundamental charge storage mechanisms and electrode materials engineering to need‐tailored device assemblies, are the key. Over the last few years, significant achievements in metal oxide and hydroxide–based aqueous supercapacitors have been reported. This work discusses the most recent achievements and trends in this field and brings into the spotlight the authors' viewpoints.

## 1 Introduction

Energy consumption has been continuously increasing and presently energy supply poses important challenges. The International Energy Agency report (2017) revealed that the worldwide energy consumption in 2015 was 9384 Mtoe (million tonnes of oil equivalent; 1 toe = 11.63 MWh), which was about 108% and 203% higher than the values in 2010 and 1973, respectively.^1^ Energy demand is expected to escalate by more than two times in 2050 and more than three times by the end of the 21st century.^2^ Fossil fuels are the main energy production source and have supplied 81.6% of global energy in 2015, but the associated greenhouse gas emission, in particular CO_2_, has raised serious environmental concerns.^3^ CO_2_ emissions increased from 15 548 Mt in 1973 to 30 326 Mt in 2010 and to 32 294 Mt in 2015, causing global warming, a major environmental issue.^1^ As a consequence, sustainable and renewable energy sources are acknowledged as central theme in the 2030 Agenda for Sustainable Development of United Nations, which underpins the economic and social roadmaps and the strategic plan of Department of Energy.^2^, ^4^ Presently, the world is assisting very fast growth of renewable energy sources; for example, hydropower increased from 1296 TWh in 1973 to 3978 TWh in 2015, wind electricity production increased from 104 TWh in 2005 to 838 TWh in 2015, and photovoltaic energy increased from 4 TWh in 2005 to 247 TWh in 2015.

Decentralized renewable energy production and its intermittency cause mismatch between energy supply and energy demand, a fact that results in energy waste and poor efficiency. Thus, energy storage is essential in the energy chain, and must be integrated with energy production to balance and stabilize energy mismatch by storing and supplying energy on demand. Moreover, voltage fluctuations in renewable energy supply require energy storage systems with fast response time, particularly for grid frequency and peak shaving. Besides grid applications, high energy and high power density energy storage devices are also essential to supply off‐grid applications such as electric vehicles, microelectronics, medical devices, and portable electronic devices. A wide array of energy storage systems tailored to the application needs are being developed, such as magnetic, electrochemical, chemical, cryogenic, mechanical, and thermal systems.^5^ Among those, supercapacitors, superconducting magnetic energy storage (SMES), and flywheels display quick response time, ranging from few milliseconds to few seconds,^6^ being the prime choice for high‐power requirements. However, SMES impose high operational costs and flywheels are bulky in size and, as a consequence, supercapacitors are becoming the most promising energy storage solutions for applications requiring high power.

For supercapacitors based on carbon electrodes, fast double‐layer charging provides high specific power (above 10 kW kg^−1^) and long operational life.^7^ However, the charge storage mechanism, essentially based on adsorption, is limited by the electrode material surface area, resulting in rather low specific energy, typically below 10 Wh kg^−1^ (3–30 times lower compared to batteries), and high self‐discharge rate.^8^ Improvement of the energy density of supercapacitors, while maintaining their high power response, is nowadays a major challenge. Charge storage in pseudocapacitive metal oxides such as cobalt oxides, RuO*_x_*, MnO_2_, and V_2_O_5_ is governed by Faradaic charge storage processes that involve sub‐valence state changes and enabled the development of high rate and energy density supercapacitors. For example, RuO_2_ and MnO_2_ display theoretical specific capacitance values above 1000 F g^−1^ in working potential windows of 1 V. More recently, hybrid supercapacitors employing redox‐active materials, which store charge via reversible redox reactions and display hybrid charge storage mechanisms, different from double‐layer charging and pseudocapacitance, are able to deliver high capacity and are considered efficient solutions to enhance energy density. Hybrid supercapacitor designs include cell assemblies in asymmetric configurations and make use of redox‐active electrodes for hybrid charge storage.

Presently, redox‐active transition metal compounds, in particular oxides and hydroxides of different metals such as Ni, Co, Fe, Ti, V, Mo, and Nb, are emerging as promising materials for electrodes used in hybrid supercapacitors.^9^ In these materials, metal sites act as redox‐active centers and sustain processes involving different valence states, a property that enables increased charge storage capacity. Moreover, since the valence changes of those compounds occur at different potentials, there is a wide choice of metal oxide/hydroxide materials that can be combined to assemble hybrid supercapacitors displaying optimal performance. For instance, in aqueous electrolytes, valence changes of Ni^2+^/Ni^3+^ and Co^2+^/Co^3+^ occur in a potential range of ≈0–0.6 V, while those of Mn^3+^/Mn^4+^ occur in the range of ≈0–1 V and those of Fe^2+^/Fe^3+^ occur in the range of −1 to 0 V.^10^ Metal oxides and hydroxides present different crystalline structures, either with open tunnels or with open interlayers, which enable ion insertion and extraction during the redox reactions and provide charge storage both in bulk sites and in surface sites. Some hydroxides contain intercalated anions for charge balancing in their layered structure; these anions are exchangeable, providing extra routes for tailoring the charge storage properties of the electrode material.

Aqueous electrolytes including acid, neutral, or alkaline electrolytes and nonaqueous electrolytes, such as organic or ionic liquids, are currently being utilized for supercapacitors.^11^ The electrolyte nature largely determines the working voltage of supercapacitors that in nonaqueous organic electrolytes can reach up to 3 V and in ionic liquids electrolytes can reach even higher values.^12^ The charge capacity increases with voltage; thus, wider working voltage obtained in nonaqueous electrolytes can support the development of supercapacitors with higher capacity. However, nonaqueous electrolytes suffer from low ionic conductivity (10–100 mS cm^−1^ for organics and <10 mS cm^−1^ for ionic liquids), leading to decreased power response. Moreover, the large molecular sizes of organic electrolytes decrease the capacitance and generally nonaqueous electrolytes bring higher costs (including high‐cost purification of water content to 3–5 ppm in organic electrolytes) and require controlled atmosphere for operation.^13^ Organic electrolytes are also toxic and flammable, raising serious safety issues during operation and disposal. Despite these serious drawbacks, current commercial supercapacitors use nonaqueous electrolytes, taking advantage of wider working voltages. Presently, supercapacitors based on aqueous electrolytes are part of many electrochemical energy storage agendas because of several advantages compared to nonaqueous electrolytes. Aqueous electrolytes present high ionic conductivity, nearly 1 S cm^−1^, which is two orders of magnitude above organic and ionic liquid electrolytes, and the hydrated ion sizes are smaller than organic, achieving higher capacity.^14^ Moreover, aqueous electrolytes are low cost, safe, and easy to handle. Although the working voltage in aqueous electrolytes (aqueous supercapacitors) is limited within the water splitting window, theoretically 1.23 V, by functionalization and engineering of the electrode materials, it is possible to widen the redox potential ranges and to expand the overpotential for water splitting. Thus, aqueous supercapacitors with working voltages up to 2.6 V have been reported.

**Figure 1:**
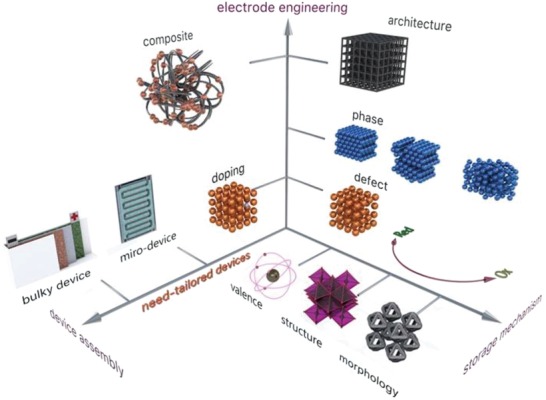
A brief scheme of relevant research streams in the field of aqueous electrochemical capacitors including charge storage mechanisms, electrode materials engineering, and functional and tailored device assemblies.

The storage capability of supercapacitors is governed by the electrode material and the charge storage mechanisms that determine the charge transfer or charge accumulation processes at electrode–electrolyte interface. Charge storage performance, response rate, and operation lifetime can be tailored by different engineering and functionalization routes that lead to optimal material physicochemical properties such as active charge storage sites, conductivity, and redox activity. The ultimate research frontier is to develop high energy density supercapacitors to fill the energy gap between supercapacitors and batteries, while keeping high power and long cycling lifetime. Functional electrodes assembled in devices tailored to the real need specificities are presently at the research forefront. The three research streams leading to the latest advances on supercapacitors are overviewed in **Figure** **1**.

In this review, authors aim at addressing the most recent research advances in metal oxide and hydroxide–based aqueous supercapacitors, spanning from fundamental charge storage studies and electrode materials engineering to need‐tailored device assemblies. Note that this review will not cover supercapacitor basics that readers may find in many dedicated reviews, perspectives, or books; instead, it highlights the latest materials functionalization and engineering trends, the respective charge storage mechanisms, and need‐tailored assemblies.

## 2 Charge Storage Mechanisms

The charge storage mechanisms generally accepted to explain the electrochemical behavior of metal oxide and hydroxide electrodes in aqueous electrolytes are based on valence changes of the metallic species through Faradaic proton/alkali metal intercalation/deintercalation, surface adsorption/desorption,^15^ or surface redox reactions with anions.^16^ Some typical examples are highlighted below

(1) $${\mathrm{RuO}}_{2}+xH^{+}+xe^{-}↔{\mathrm{RuO}}_{2-x}{\left(\mathrm{OH}\right)}_{x}$$

(2) $${\mathrm{MnO}}_{2}+C^{+}+e^{-}↔\mathrm{MnOOC}$$

(3) $$\mathrm{NiO}+{\mathrm{OH}}^{-}↔\mathrm{NiOOH}+e^{-}$$

(4) $${\mathrm{Co}}_{3}O_{4}+H_{2}O+{\mathrm{OH}}^{-}↔3\mathrm{CoOOH}+e^{-}$$

(5) $$\mathrm{Co}{\left(\mathrm{OH}\right)}_{2}+{\mathrm{OH}}^{-}↔\mathrm{CoOOH}+H_{2}O+e^{-}$$

(6) $$\mathrm{CoOOH}+{\mathrm{OH}}^{-}↔{\mathrm{CoO}}_{2}+H_{2}O+e^{-}$$

(7) $$\mathrm{Ni}{\left(\mathrm{OH}\right)}_{2}+{\mathrm{OH}}^{-}↔\mathrm{NiOOH}+H_{2}O+e^{-}$$

where C^+^ is an alkaline ion or a proton in the electrolyte.

Although these mechanisms are widely accepted, they are rather general and lack detail on how the physicochemical properties of the electrode material change during the charge storage and charge release processes. Relevant materials' properties include morphology, structure, and valence states (Figure 1, storage mechanism axis); their changes during the electrochemical processes that store and release charge govern electrode performance such as charge capacity, response rate, and operational lifetime. Thus, understanding such changes is essential to design electrodes tailored to meet the specificities of the final application.

Preliminary understanding of valence and local structure changes of MnO_2_ electrodes has been obtained using in situ X‐ray absorption near‐edge structure (XANES) and extended X‐ray absorption fine structure (EXAFS).^17^ The shift of Mn K‐edge peak in XANES revealed that MnO_2_ valence gradually increased from +3.23 to +3.95 when charging, and gradually decreased to 3.27 after discharging in aqueous 2 m KCl electrolyte.[[qv: 17a]] The irreversibility of Mn valence states during charge storage is responsible for capacity decay after cycling; however, reversibility of the valence states and the local structure of MnO_2_ depend upon the working potential window.[[qv: 17b]] The formation of low‐valence states after discharge resulted in Mn dissolution, leading to capacity decay. Recently, several in situ*/*operando observations addressing morphological, valence, and structural changes of metal oxides/hydroxides have been reported and have provided new insights into the charge storage mechanisms of metal oxide/hydroxide electrodes in aqueous electrolytes. Thus, in the following subsection the most recent achievements on important metal oxides and metal hydroxides are overviewed.

### 2.1 Metal Oxides

#### 2.1.1 Mn Oxides

**Figure 2:**
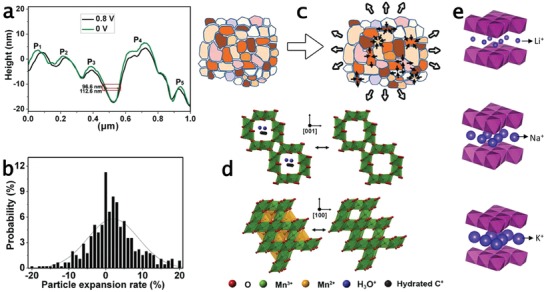
Charge storage mechanisms of γ‐MnO_2_ revealed from a–c) in situ AFM and d,e) operando Raman spectroscopy. a) AFM height profiles at 0 and 0.8 V; b,c) MnO_2_ particle expansion and its localized contraction from the proton deintercalation process. d) Top: proton and cation deintercalation in the MnO_2_ lattice; bottom: transformation from Mn^2+^ to Mn^3+^. e) Influence of cation size on MnO_2_ structural changes. a–c) Reproduced with permission.^18^ Copyright 2013, John Wiley and Sons. d) Reproduced with permission.^22^ Copyright 2014, Elsevier Ltd. e) Reproduced with permission.^23^ Copyright 2015, American Chemical Society.

Morphological and mechanical changes of γ‐MnO_2_ during the charge/discharge process in 1 m KCl were studied by in situ atomic force microscopy (AFM) and nanoindentation built in within AFM.^18^ This study evidenced the MnO_2_ particle contraction/expansion during the charge/discharge process (**Figure** **2**a,b). The expansion was inhomogeneous (Figure 2b), and localized contractions of grains were also observed (Figure 2c). Pores among the particles were shrunken during particle expansion and localized grain contractions and interparticle pore shrinkage compensated such expansion. This behavior was explained by proton intercalation, reaction (2), which induced lattice expansion and changes in Mn ionic radius upon valence changes that increased from 0.530 Å for Mn^4+^ to 0.645 Å for Mn^3+^, in the discharge process. In situ nanoindentation revealed softening of MnO_2_ by proton intercalation, due to reduction of the elastic modulus and hardness of MnO_2_ after discharging, in agreement with particle expansion. This contraction/expansion during the charge/discharge process rendered for morphological changes, which are often observed in metal oxide–based electrodes after cycling. Thus, it is worth stating that MnO*_x_*‐based electrodes with porous structures are the key to overcome contraction/expansion‐related drawbacks and to design active materials displaying increased cycling stability.

Mn valence changes in different MnO_2_ polymorphs have been recently reported and, for example, Mn valence state changes in δ‐MnO_2_ nanosheets were investigated by in situ X‐ray absorption spectroscopy (XAS) in the near‐edge region.^19^ The as‐prepared δ‐MnO_2_ displayed Mn valence state of +3.16, which is lower than the theoretical valence state of +4 due to the presence of preintercalated cations, which attracted electron from oxygen sites in good agreement with results published elsewhere.[[qv: 17a]] After immersion in 0.5 m Na_2_SO_4_, the Mn valence state decreased to +3.09, effect explained by intercalation of Na^+^. The Mn valence state progressively increased to +3.23 when charging up to 0.6 V versus saturated calomel electrode (SCE) and continuously decreased to +3.05 upon discharging to 0 V versus SCE. The changes in the valence states were due to deintercalation and intercalation of cations on charging and discharging, respectively. Similarly, Mn valence state of K–birnessite MnO_2_ nanosheets increased from +3.01 to +3.12 upon charging from 0 to 0.8 V versus SCE and decreased to +3.01 upon discharging to 0 V versus SCE.^20^ Overcharging in the oxygen evolution region resulted in irreversible valence changes.^21^ These results were confirmed by different reports addressing Mn valence changes in MnO_2_ electrodes during the charge storage and release processes. It has been suggested that the valence changes are not influenced by MnO_2_ polymorphs, but it has been evidenced that valences of +3.23 and +3.12 at full charge state are still far from full oxidation (+4). Summarizing, the reported work demonstrates that there is still insufficient exploration of the full Mn valences for charge storage purposes, a research line that is likely to be pursued in the near future, thanks to the attractiveness of Mn oxides as charge storage materials.

Structural changes of α‐Mn_0.98_O_2_ during the charge/discharge process in Na_2_SO_4_ were monitored by operando Raman spectroscopy.^22^ The results revealed structural transformations due to insertion of hydrated Na^+^ in the tunnel of MnO_2_, in agreement with the valence changes proposed elsewhere.^19^ The transformation from MnO_2_ to Mn_3_O_4_ phase was also noticed, resulting in the coexistence of Na–MnO_2_ and Mn_3_O_4_ after immersion in the electrolyte. The hydrated proton intercalation into the tunnel and hydrated Na^+^ deintercalation occurred when the electrode was slowly charged to 0.06 V, resulting in the formation of MnOOH. The proton and Na^+^ were fully deintercalated when charging to 0.6 V (Figure 2d, top). At higher charging voltage, from 0.6 to 1 V, Mn_3_O_4_ was transformed into MnO_2_ phase, phenomenon associated with deintercalation of Mn^2+^ at octahedral sites in Mn_3_O_4_ (Figure 2d, bottom). During the discharge process, from 1 to 0 V, reversible reactions occurred, associated with Mn^2+^ intercalation in the voltage range from 1 to 0.6 V and proton and Na^+^ intercalation/deintercalation in 1 × 1 tunnel under the voltage range from 0.6 to 0 V.

The effect of different cation sizes (Li^+^, Na^+^, and K^+^), in aqueous electrolytes, on the structural changes and charge storage performance of MnO_2_ electrodes was also studied by operando electrochemical Raman spectroscopy.^23^ In the high charge state of MnO_2_, water molecules intercalated in the tunnels/interlayer, resulting in structural similarity in aqueous electrolytes with different cations. The charge storage mechanism was similar when different cations were used, and during the discharge process, cations were intercalated into the tunnels, replacing water intercalation. The smaller ionic radius of cations, K^+^ (1.37 Å), Na^+^ (0.99 Å), and Li^+^ (0.59 Å), compared to water (2.8 Å) resulted in decreased interlayer spacing during discharge. The different sizes of cations intercalated into MnO_2_ tunnels are responsible for structural changes during the charge/discharge process and increased cation size augments the interlayer distance (Figure 2e). Structural polarizability (vibration mode polarizability) changes were noticed when the size of the intercalated cation increased. There was a polarization in the Mn—O vibration along the chains of the MnO_2_ framework compared to the symmetric stretching vibration of Mn—O bond in the MnO_6_ octahedra. Increased cation size also decreased the Jahn–Teller distortion when the electrode was fully discharged. Higher specific capacitances and rate performance were obtained with smaller cation size due to the favored cation intercalation process, which reduced charge transfer resistance, diffusion resistance, and diffusion time constant. However, electrolytes with smaller cation size (e.g., Li^+^) resulted in poorer charge–discharge cycling stability. This result was due to the high structural changes (Jahn–Teller distortion) that induced faster degradation of the electrode when it was cycled in electrolytes with smaller size cations. Summarizing, it is clear that electrolyte cations play a pivotal role in defining the valences changes and electrode capacity and stability. The optimal combination in terms of Mn oxides and electrolyte composition still represents an important gap in the state of the art and further studies are very necessary to better clarify this issue.

Valence state and structural changes of Mn_3_O_4_ during charge–discharge processes in 1 m Na_2_SO_4_ were examined by in situ XANES and X‐ray diffraction (XRD).^24^ When charging the Mn_3_O_4_ electrode from 0 to 1.2 V versus Ag/AgCl, the Mn valence state was stable in the potential range of 0–0.75 V, but increased in the potential range of 0.75–1.2 V. This discontinuous valence change was different from the continuous change in MnO_2_ discussed above, and can be related to redox reactions occurring at high potentials. In situ XRD during the charge–discharge revealed the spinel lattice contraction–expansion, which correlated well with the oxidation–reduction processes; no phase changes were observed. The charge storage mechanisms of the Mn_3_O_4_ electrodes were also detailed by operando Raman spectroscopy^25^ and, contrarily to the previous report, an irreversible transformation from Mn_3_O_4_ to MnO_2_ phase and Na^+^ and Mn^2+^ (extracted from tetrahedral sites) intercalation into the defects or tunnel sites of the MnO_2_ in the first charge were noticed (Equation (8))

(8) $${\mathrm{Mn}}_{3}O_{4}\to {\mathrm{Na}}_{x}{\mathrm{MnO}}_{2}\cdot nH_{2}O$$

Discharge associated with the intercalation of Na^+^ cations in the tunnel induced an electrochemical transformation of the MnO_2_ phase. Studies on the transformed MnO_2_ revealed the softening of the MnO_2_ lattice due the reversible deintercalation of Na^+^ and intercalation of H_2_O due to the electrostatic repulsion–induced expansion of interlayers when charging. The partial reversible oxidation of Mn^2+^ in the interlayers to Mn^3+^ was also proposed, resulting in partial formation of Mn_3_O_4_ after complete deintercalation of Na^+^

(9) $${\left({\mathrm{MnO}}_{2}{\;}^{2-}{\mathrm{Mn}}^{2+}\right)}_{\mathrm{interlaminar}}-e^{-}↔{\mathrm{Mn}}^{3+}{\mathrm{MnO}}_{2}{}^{2-}$$

#### 2.1.2 V Oxides

V_2_O_5_ has commonly been thought to store charge via cation intercalation/deintercalation reactions and displayed good pseudocapacitive response, but its poor cycle life is still hindering its use. Thus, understanding its charge storage mechanism is essential to develop new V_2_O_5_ electrodes with improved performance. In situ XRD tracking of structural changes in K*_x_*V_2_O_5_ electrodes during charge–discharge in KCl electrolyte revealed that the (001) plane lattice expanded on charging and contracted on discharging.^26^ The charging was related to K^+^ deintercalation from (001) interlayers, which weakened the interaction of K^+^ with negatively charged [VO_6_] octahedra, leading to expansion of (001) interlayers. On discharging, the reverse process led to the lattice contraction. Structural and mass changes of α‐V_2_O_5_ nanowire electrodes during the charge storage and release processes in aqueous 1 m Na_2_SO_4_ were studied by operando Raman spectroscopy.^27^ Structural changes of α‐V_2_O_5_ during charge–discharge cycling revealed that no phase changes occurred and that V—O—V and V—O bond lengths were changed. These changes could be associated with the expansion/compression of (001) planes during the charge/discharge process as a result of Na^+^ (or their hydrated form) deintercalation/intercalation into V_2_O_5_ lattice, as revealed by previous work.^26^ In situ electrochemical quartz crystal microbalance showed the mass loss and the mass increase during the charge/discharge processes, which could be due to the reversible mass change from Na^+^ (or their hydrated form) deintercalation/intercalation reactions. The mass of V_2_O_5_ was reduced after one charge/discharge cycle when compared to the initial mass, suggesting an irreversible mass loss of the electrode due to dissolution into the electrolyte, which resulted in poor cycle life. The existing studies highlight the potential of this material and anticipate an important gap related to the need of developing solutions to prevent dissolution of the V_2_O_5_ active material during the charge/discharge cycling, to increase its electrochemical performance.

#### 2.1.3 Co Oxides

Co oxides have attracted enormous attention as active materials for supercapacitors, providing charge storage, thanks to Faradaic and non‐Faradaic processes, and have been studied since many years. Different cobalt oxides with different morphologies are easy to synthesize and provide very high theoretical capacitances. In alkaline electrolytes, Co_3_O_4_ is converted into CoOOH, which in turn can be converted into CoO_2_. Despite its high theoretical capacitance, in practice values achieved are much lower and the main reason has been attributed to the decreased conductivity and formation of parasitic species over cycling. It is worth noting that sometimes reported capacitances are miscalculated because of the battery‐type signature of this material. Moreover, it has been noticed that the lifetime of cobalt oxide electrodes has been limited by expansion/contraction phenomena. The design of different high surface area morphologies has been the main route to overcome these drawbacks and the literature offers a variety of cobalt oxides produced by different routes such as Co_3_O_4_ nanowires, nanosheets, microspheres, nanospheres, hollow spheres, thin films, and many others. It has been shown that the electrochemical performance depends on the synthesis route, surface area pore size, and nature of the electrolyte. Despite all the advances and popularity of this material, the active potential window in alkaline electrolytes is typically limited to ≈0.5 V, an issue that limits its specific capacitance and its use. The strategy to overcome these drawbacks is nowadays focused on doping or combining cobalt oxides with other transition metal oxides or modification with heteroatoms, a research line that may enable the design of supercapacitor electrodes with increased stability in wider potential windows. Despite the research interest, scarcity of cobalt and its actual high price are limiting practical applications of Co‐based electrodes for charge storage in both the battery and supercapacitor field.

### 2.2 Metal Hydroxides

#### 2.2.1 Ni Hydroxides

**Figure 3:**
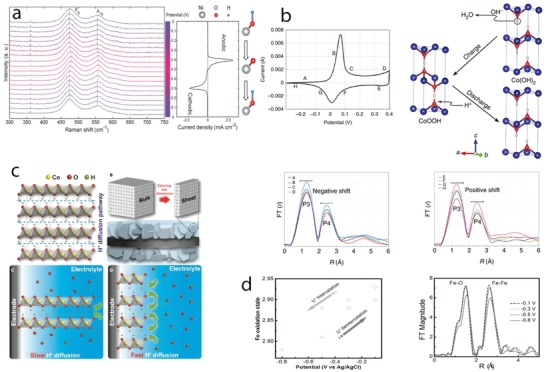
a) Operando electrochemical Raman spectra of NiOOH under charge–discharge processes in KOH electrolyte and O—H bond formation and breaking during the redox reactions of NiOOH. b) In situ EXAFS spectra of Co(OH)_2_ during charge–discharge processes recorded at different potentials noted in the cyclic voltammogram; P3 and P4 correspond to Co—O and Co—Co shells; H^+^ deintercalation/intercalation during Co(OH)_2_ ↔ CoOOH conversion. c) Diffusion pathway of H^+^ into layered Co(OH)_2_ structure; CoO_2_ plane orientation of Co(OH)_2_ nanoplatelets influences H^+^ diffusion: slow diffusion with CoO_2_ parallel to the platelets and fast diffusion with CoO_2_ vertical to the platelets. d) Fe valence state changes with potentials and in situ EXAFS spectra when charging from −0.8 to −0.1 V. a) Reproduced with permission.^28^ Copyright 2016, John Wiley and Sons. b) Reproduced with permission.^30^ Copyright 2017, Springer Nature. c) Reproduced with permission.^31^ Copyright 2017, Elsevier Inc. d) Reproduced with permission.^32^ Copyright 2014, John Wiley and Sons.

Structural changes of NiOOH electrodes in alkaline electrolytes have been studied by operando Raman spectroscopy and the results revealed that no relevant phase changes occurred during the charge/discharge processes.^28^ Structural evolution mainly arose in the potential ranges where redox reactions occurred (0.35–0.5 V during the charge process and 0.5–0.3 V during the discharge process) (**Figure** **3**a). The charge process shortened the Ni—O bonds, which resulted from stiffening of Ni—O stretching vibration and Ni—O bending vibration modes; an increase of vibrational polarizability to Ni—O stretching modes also occurred after charging, as revealed by blueshifts of E_g_ and A_1g_ modes and an increase in A_1g_/E_g_ intensity ratio in Figure 3a. This process was reversible, leading to the lengthening of Ni—O bond, softening of Ni—O stretching and bending vibrations, and reduction of vibration polarizability. Changes in oxidation states were also noticed at high charge/discharge conditions. This was assigned to the reversible transformation of Ni^3+^ to Ni^4+^ as widely discussed in the literature. The structural evolution and oxidation state changes have been attributed to consecutive breaking and formation of O—H bonds during the reversible redox reactions (Figure 3a). Electrolyte cations (Li^+^, Na^+^, or K^+^) do not seem to influence the structural changes during charge storage. Layered NiOOH can host a number of cations in the interlayer. However, only minor structural disorders have been noticed after cation intercalation/deintercalation, and it can be considered that their contribution to the charge storage capacity was not relevant. The charge storage capacity varied with Ni—O bond length, softening/stiffening of Ni—O vibration, and their polarizability.

Merrill et al. suggested that oxygen redox chemistry could also contribute to the charge storage besides the commonly accepted mechanisms based on valence changes of metallic ions.^29^ Using operando Raman spectroscopy and thermodynamic analysis, they proposed a charge storage mechanism for Ni(OH)_2_ and hydrated NiO, involving the oxidation/reduction of Ni^2+^/Ni^3+^ and the oxygen redox chemistry instead of Ni^4+^ involvement. This effect is still far from being well understood and further studies are still crucial to clarify how the oxygen redox chemistry contributes to the high charge storage capacity of Ni(OH)_2_ electrodes. A major drawback on the application of Ni hydroxides is the lack of conductivity over cycling, an issue that requires construction of nanoporous and nanosized architectures to enhance both electron transfer and ion diffusion.

#### 2.2.2 Co Hydroxides

Co(OH)_2_ is a promising charge storage electrode material working in alkaline electrolyte, which displays high specific capacity, high response rate, and long cycle life. However, the intrinsic properties and mechanisms underlying their good charge storage capability are still very less understood. Recently, in situ electrochemical XAS and density functional theory (DFT) calculations have been carried out during the charge storage and release process on Co(OH)_2_ platelet electrode.^30^ The shifts of Co K‐edges in XANES spectra revealed that Co valence state increased on charging from −0.3 to 0.4 V and decreased upon discharging. The Co valence states reversibly changed as evidenced by the same absorption Co K‐edge positions after charge/discharge processes. The XAS oscillation pattern of the electrode after discharging showed no changes, suggesting negligible structural changes. Only some small adjustments of atoms occurred, involving the decrease of Co—O and Co—Co bond lengths and structural disorder on charging and vice versa on discharging, as revealed by the negative shifts and increased intensity of Co—O and Co—Co shells in EXAFS results during charging and reverse changes during discharging (Figure 3b). The bond length variations are 0.06 and 0.22 Å for Co—O and Co—Co, respectively. DFT calculations showed that the energy difference of the deprotonated Co(OH)_2_ and ground‐state CoOOH is as low as 0.59 eV, and the activation energies for the phase transformation of Co(OH)_2_ to CoOOH and CoOOH to Co(OH)_2_ are 0.76 and 0.34 eV, respectively. This low activation energy, plus the minor structural changes for the phase transformation, explained the high power response and long cycle life of Co(OH)_2_. H^+^ deintercalation/intercalation during the phase transformation processes (Figure 3b), which is similar to charge storage in batteries, resulted in increased specific capacity. This study evidenced the concept of developing electrode materials, which can display high power response similar to supercapacitor materials, while achieving energy densities similar to battery materials. For that, structural similarity of the redox reactants is essential for high specific power and high cycle life and the deintercalation/intercalation redox reaction is essential for high specific energy.

Goodenough and coworkers explained further the results obtained for Co(OH)_2_ and extended them to Ni(OH)_2_ electrodes with structure and redox processes similar to Co(OH)_2_.^31^ While Co(OH)_2_ showed good response rate and cycling life, these properties are generally poor for its isostructural Ni(OH)_2_. The good response rate and cycle life of Co(OH)_2_ platelets were attributed to its CoO_2_ planes vertical to the large area of the platelet, providing short pathways for H^+^ diffusion for the bulk redox reactions (Figure 3c). Furthermore, the highly reversible transformation of Co(OH)_2_/CoOOH could prevent morphological breaking due to volume changes during H^+^ deintercalation/intercalation processes. This orientation was possibly due to the 3d orbital ordering on the octahedral Co^2+^ ions, which is absent in octahedral Ni^2+^ in Ni(OH)_2_. It could result in different orientation of NiO_2_ planes, rather than vertical to the outer surface of the nanostructures, which led to slow bulk redox processes and hence poor response rate and cycle life.

#### 2.2.3 Fe Hydroxide

Iron oxy hydroxide, FeOOH, displays pseudocapacitive response mainly in the negative region of the working potential window in aqueous electrolytes, being presently studied as negative electrode in asymmetric supercapacitors. However, as for other hydroxides, the charge storage mechanism of FeOOH still remains vague and many details are lacking. Preliminary work on the charge storage mechanism of nanoplatelet lepidocrocite γ‐FeOOH in 1 m Li_2_SO_4_ made use of in situ electrochemical XAS during the charge/discharge process.^32^ The results showed only the shift of Fe K‐edge during the charging and discharging of FeOOH electrode, which suggests structural similarity and oxidation state changes during the redox reactions. The Fe—O bond length in the octahedral FeO_6_ decreased when the working potential increased from −0.8 to −0.1 V versus Ag/AgCl, as shown in EXAFS results in Figure 3d, which corresponded to a change in the Fe oxidation state from +2.78 to +2.93 (Figure 3d). Fe—Fe bond length for the Fe atoms between neighboring octahedral FeO_6_ also decreased, suggesting the contraction of layered FeO_6_, which is probably related to deintercalation of Li^+^. During the Li^+^ deintercalation reaction, structural order increased as revealed by the increase of Fourier‐transformed magnitude of Fe K‐edge EXAFS (Figure 3d), corresponding to restoration of symmetric oxygen distribution in the octahedral FeO_6_. The charge storage mechanism of FeOOH was explained considering valence changes in Fe atoms, from +2 to +3, during the Li^+^ deintercalation/intercalation reaction as

(10) $$\mathrm{Fe}\left(\mathrm{III}\right)\mathrm{OOH}+\mathrm{Li}+↔\mathrm{LiFe}\left(\mathrm{II}\right)\mathrm{OOH}$$

The above discussion provides important insights into the charge storage mechanisms of metal oxide/hydroxide–based electrodes. More details about valence state changes, structural changes, and morphological changes during the operative condition of the electrodes have been reported. Important work has contributed to better understand the physicochemical changes occurring during charge storage and discharge; however, some contradictory results have also been observed and it is worth noting that during operando Raman measurements, structural changes induced by laser beam during measurements could result in the misleading results and interpretation. While many oxides and hydroxides display charge storage ability in aqueous electrolytes, only a limited number has been deeply studied and the respective charge storage mechanisms explained. Indeed, for the same oxides/hydroxides, there are different phases/polymorphs, which strongly impact the charge storage processes and mechanisms, an issue that still requires deeper studies. Moreover, the role of different electrolytes in the charge storage processes of many oxides and hydroxides is far from being well understood. Further work on these important subjects is still necessary to unveil the charge storage mechanisms in different electrolytes and to develop more reliable electrodes overcoming the drawbacks that limit the practical use of metal oxides and hydroxides as electrodes for supercapacitors.

## 3 Electrode Materials Engineering and Functionalization

Presently, most of the studies on metal oxides/hydroxides for supercapacitors focus on the design and development of electrodes that aim at achieving higher energy and power density and long‐term charge storage/release stability. On the one hand, intrinsic properties of metal oxides/hydroxides such as metal atoms, crystal structure/orientation, defect chemistries, and morphologies govern the charge storage performance. On the other hand, extrinsic materials engineering and functionalization, via atomistic doping and formation of composites with other materials, are also expected to improve electrochemical performance much further. Figure 1 (electrode engineering axis—EEA) depicts different intrinsic (right side of EEA, Figure 1) and extrinsic (left side of EEA, Figure 1) materials engineering routes to modify supercapacitor electrode properties. These properties are briefly addressed in the following parts of this section.

Concerning metal elements, cations with high number of electrons transferred during the redox reactions in aqueous electrolytes are expected to deliver high charge storage capacity. Their redox‐active potential range and overpotential for water splitting determine the working potential window. Low electronegative cations can facilitate electron transfer, improving high power density. High electronegative cations can increase the electrochemical potential of the redox reactions, resulting in high charge storage capacity. Electronegativity of metal cations can also vary the metal–oxygen bond length and ion adsorption capability, leading to different energy and power densities as suggested by the results of operando electrochemical Raman spectroscopy discussed in the previous section. Metal cations, with low molar mass, incorporated in low molar units are beneficial for high gravimetric capacity electrodes. For example, NiO (74.69 g mol^−1^) has molar mass lower than MnO_2_ (86.93 g mol^−1^) and Co_3_O_4_ (240.80 g mol^−1^), resulting in higher theoretical gravimetric capacity values.

Many metal oxides and hydroxides currently under investigation for charge storage electrodes in aqueous supercapacitors display different polymorphs. Their crystal structure affects the electronic structure, namely, bandgaps and local electron density, which in turn influence electron conductivity and the redox activity of the electrodes. Crystal structures with 2D interlayer or 1D tunnel favor bulk diffusion of electrolyte ions, while close‐packed crystal structures can support only surface redox reactions, unless structural changes occur during the charge storage/release process. Thus, structural instability during the redox charging and discharging can alter the charge storage mechanisms. Incorporating metal cations with different ionic radii into mixed metal oxides and hydroxides can induce stress/strain and change the metal–oxygen bonding in the crystals, which affect directly the redox activity. Amorphous phases can facilitate charge transfer reactions due to their weak chemical bonding and presence of more surface defects.^33^ Defect chemistries, such as cation or oxygen vacancies, affect electronic structure and can change redox activity.^34^

The theoretical specific capacitance (F g^−1^), assuming that the charge storage is constant over the working potential, can be calculated based on the number of electrons that are stored/released during the redox reactions according to the following equation

(11) $$C=\left(\frac{nF}{m}\right)\frac{X}{E}$$

where *n* is the number of electrons stored/released in the redox reactions, *F* is the Faraday constant (96 485 C mol^−1^), *m* is the molar mass of metal oxides, and *E* is the working potential window. *X* is the surface fraction (or electrode/electrolyte interface) of oxide material. This equation demonstrates that high experimental specific capacitance values can only be achieved when the oxide electrodes possess high specific surface area to maximize the number of active sites for the redox reactions. Therefore, it highlights how relevant is the specific surface area to improve the specific capacitance of the electrode. Electrode morphology can define surface area and porosity, affecting the active sites for the redox reactions.^35^ Electrolyte diffusion pathways, which influence the response rate or power, also depend on the electrode morphologies. These morphologies can be controlled by different preparation processes inducing different crystal growths and crystal orientations, leading to different exposed crystal facets. Surface energy of the different facets is varied, resulting in different redox activities and thus altering the charge storage performance. The morphologies should possess micro/nanoporosity to compensate the expansion/contraction of grain/particle, softening/stiffening of chemical bonds during the charge–discharge cycling, and increase the ionic diffusion of electrolyte ions into the electrodes. Nanosizing and nanostructuring are thus crucial routes to prepare electrode materials with enhanced energy storage capacity.^36^

The control of intrinsic properties in metal oxide and hydroxide electrodes is the key route to optimize electrochemical performance. Intrinsic properties such as low electron conductivity of both oxides and hydroxides and structural instability of hydroxides are not easy to improve via intrinsic materials engineering. Thus, the development of extrinsic materials engineering and functionalization approaches, via cation or anion doping, or compositing with other materials, enables new routes to enhance the charge storage performance of the electrodes further. For example, hybrid electrodes composed of electron conducting channels coated with high redox‐active metal oxides and hydroxides display boosted storage performance via extrinsically increased electron conductivity and surface area. Composites of metal oxides and hydroxides with conducting metals or carbon‐based nanomaterials are currently the main research stream to improve electrodes' performance.

Intrinsic and extrinsic materials engineering have been mainly controlled by the routes used to prepare the electrode materials as well as by the postprocessing methods. Next, in this section, recent advances in electrode materials engineering and functionalization via intrinsic and extrinsic approaches towards enhanced electrochemical performance will be highlighted. Note that for metal oxides or hydroxides displaying battery‐like behavior, the specific charge storage capacity should be presented as C g^−1^ or mAh g^−1^ (or per cm, cm^2^, or cm^3^) because the specific charge is not constant over the working potential window. Since many reports have presented specific charge capacity as F g^−1^, in this discussion, the values will be used as reported in the original literature. The main research trends have been focused on strategies to obtain high gravimetric energy density electrodes; however, for practical application of supercapacitors, length, areal, and volumetric capacities are becoming important metrics as reported in several studies. These will also be discussed in the context of the intrinsic and extrinsic materials engineering approaches.

### 3.1 Intrinsic Materials Engineering

#### 3.1.1 Crystal Engineering

**Figure 4:**
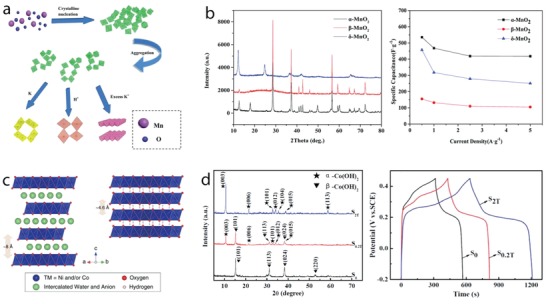
a) A schematic diagram illustrating the influence of cations (H^+^ and K^+^) on the formation of different MnO_2_ polymorphs; b) XRD patterns of α‐MnO_2_ (*C*_K+_ > *C*_H+_), β‐MnO_2_ (*C*_H+_ > *C*_K+_), and δ‐MnO_2_ (excess of K^+^) and their specific capacitance values at different specific currents. c) Crystal structures of α (left) and β (right) phases of Ni or Co hydroxides; d) XRD patterns of Co(OH)_2_ polymorphs prepared at different applied magnetic fields and their charge–discharge curves at a specific current of 1 A g^−1^. a,b) Reproduced with permission.^38^ Copyright 2015, Royal Society of Chemistry. c) Reproduced with permission.^40^ Copyright 2016, John Wiley and Sons. d) Reproduced with permission.^41^ Copyright 2017, Royal Society of Chemistry.

*Phase Engineering*: Manganese dioxide, MnO_2_, crystallizes in different crystal polymorphs, such as α, β, γ, δ, and λ forms, and various of these polymorphs have been studied as charge storage electrodes for supercapacitors. These different crystal polymorphs are based on different arrangement of MnO_6_ octahedral units, forming 1D tunnel structures (α phase, 2 × 2 open tunnels; β phase, 1 × 1 open tunnels; and γ phase, 1 × 2 open tunnels), 2D interlayer structure (δ phase), and 3D spinel structure (λ phase), which display different electrochemical activity. Thus, optimization of crystal polymorphs of MnO_2_ is being considered an important route to achieve higher‐performance electrodes. Earlier work on crystal engineering of MnO_2_ has been reported in the last decade^37^ and the outcomes revealed that large tunnel size in α phase and high interlayer distance in δ phase support better intercalation/deintercalation reactions and display higher capacitance values compared to other phases. Although these results provide some initial insights into how crystal structures of MnO_2_ would affect specific capacitance, the capacities obtained are modest (200 F g^−1^). Moreover, the materials chemistry and engineering routes proposed to control MnO_2_ polymorphs and to optimize their electrochemical performance are still little understood. Recently, MnO_2_ with α, β, and δ polymorphs have been synthesized by controllable hydrothermal reactions by varying the concentration of K^+^ (*C*_K+_) and H^+^ (*C*_H+_) cations in the electrolyte (**Figure** **4**a).^38^ The α phase formed when the *C*_K+_ is higher than *C*_H+_, the β phase formed when *C*_H+_ is higher than *C*_K+_ and the δ phase formed in excess of K^+^, as shown in a schematic diagram and XRD patterns in Figure 4b. K^+^ stabilized the formation of 2 × 2 tunnels during MnO_2_ dissolution–recrystallization processes and excess of K^+^ induced the formation of 2D interlayer by destructing the 2 × 2 tunnels. α and δ phases displayed, respectively, specific capacitance values of 535 and 464 F g^−1^, which are higher than the 155 F g^−1^ of the β phase due to increased intercalation reactions in 2 × 2 tunnel and interlayer structure compared to 1 × 1 tunnel structure, when the materials are tested in 1 m KOH electrolyte (Figure 4b, right).

Charge storage performance of TiO_2_ nanotube arrays with different polymorphs has been studied by thermal transformation of the anatase phase to the rutile phase.^39^ Rutile TiO_2_ nanotubes displayed capacitance values of 2.6 mF cm^−2^ at 1 mV s^−1^, which are higher than those of anatase phase due to higher conductivity and presence of Ti^+3^ after forming rutile phase. Although many metal oxides such as MoO_2_, MoO_3_, V_2_O_5_, and Fe_2_O_3_ exist in different polymorphs, only scarce work discussing controllable crystal phase engineering has been reported up to date.

Ni(OH)_2_ and Co(OH)_2_ display isostructures composed of two different polymorphs: α and β (Figure 4c).^40^ α phases crystallize in the hydrotalcite structure, with positively charged Ni(OH)_2_ or Co(OH)_2_ layers, balanced by intercalated anions and display a high interlayer spacing of ≈8 Å. β phases crystallize in brucite‐type hydroxide, with hexagonal close‐packed structure, and show an interlayer spacing of ≈4.6 Å. The higher interlayer spacing in the α phase supports intercalation redox reactions, making this phase theoretically very interesting for supercapacitor electrodes. Nevertheless, the α phase is a metastable one, being quickly converted into the β phase in alkaline media. Thus, controlling the formation of different polymorphs and comparing their electrochemical response is very challenging. Recently, the formation of α‐Co(OH)_2_ and β‐Co(OH)_2_ has been selectively controlled by magnetic field–driven hydrothermal synthesis.^41^ The α‐Co(OH)_2_ was formed under an applied magnetic field of 2 T at 180 °C, the mixed α and β phases were formed under 0.2 T, whereas β‐Co(OH)_2_ was formed in the absence of the magnetic field, revealing the magnetic field–induced phase transformation, as shown in XRD patterns (Figure 4d, left). α‐Co(OH)_2_ phase showed specific capacity values of 885 F g^−1^ at 1 A g^−1^, which increased compared to the value of 388 F g^−1^ at 1 A g^−1^ of β‐Co(OH)_2_ (Figure 4d, right). α‐Ni(OH)_2_ and β‐Ni(OH)_2_ have also been selectively grown by varying the reaction temperature during the hydrothermal process.^42^ Some studies have focused on the preparation of the α phase due to its higher theoretical redox activity compared to the β one. Novel approaches have been proposed to produce α‐Ni(OH)_2_ such as bioinspired synthesis via two ion chamber reactors of Ni^2+^ cations and OH^−^ anions separated by a Nafion membrane.^43^ This route produced α‐Ni(OH)_2_, which delivered specific capacity of 2090 F g^−1^ at a specific current of 2 A g^−1^. The solvothermal process produced layered (4–5 nm in thickness) α‐Ni(OH)_2_ nanosheets that delivered specific capacity of 2064 F g^−1^ at 2 A g^−1^.^44^ It has been demonstrated that the β phase could also deliver high charge storage capacity. For example, microwave‐assisted hydrothermal synthesized β‐Ni(OH)_2_ presented specific capacity of 1566 F g^−1^ at 1 A g^−1^,^45^ and oriented‐attachment synthesized single‐layer β‐Co(OH)_2_ presented specific capacity of 2028 F g^−1^ at 5 A g^−1^.^46^ Despite these advances, the cycle life of Ni(OH)_2_ polymorphs is still very problematic compared to Co(OH)_2_ polymorphs, a consequence of the structural instability discussed in the previous section.

Amorphous metal oxides and hydroxides have also been investigated for aqueous supercapacitor electrodes. For example, amorphous Ni–Co–Fe hydroxide and Ni hydroxide have been prepared by different routes and display good charge storage performance.^47^ However, for these materials no relevant structural–electrochemical relationship has been reported up to date, to the best of our knowledge, and further research is required to understand the charge storage behavior.

**Figure 5:**
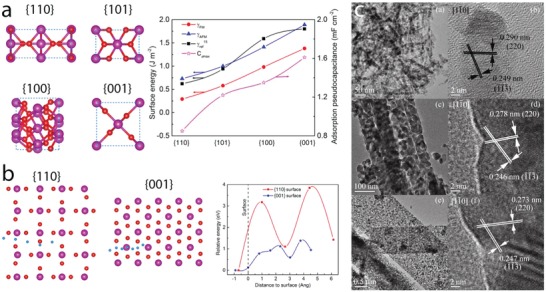
a) Left: top view of {110}, {101}, {100}, and {001} surfaces of β‐MnO_2_; right: surface energy and adsorption pseudocapacitance. b) Proton diffusion through {110} and {001} surfaces (side view) and the diffusion energy barrier. c) TEM) and high‐resolution TEM images of Co_3_O_4_ nanorods (top), nanobelts (middle), and nanosheets (bottom) show the formation of high‐energy {110} facets. a,b) Reproduced with permission.^48^ Copyright 2017, American Chemical Society. c) Reproduced with permission.^49^ Copyright 2011, Springer Nature.

*Crystal Facet Engineering*: The contribution of surface activity of the electrode materials to the redox reactions is closely related to their charge storage performance. From the crystal point of view, surface activity is associated with crystal facets, where different atomic arrangements on the outer surface alter surface energy and redox activity. Consequently, understanding the activity of different facets and controlling the formation of highly active facets are important goals to enhance the electrode performance. Whereas highly active facets play a main role in surface activity of materials with compact crystal structures such as NiO, Co_3_O_4_, and NiCo_2_O_4_, metal oxides and hydroxides with large open tunnels or interlayers, which support the bulk (intercalation) redox reactions, can also be influenced by the orientation of the open tunnels or interlayers to the outer surface. Surface energy of four low‐index crystal facets of β‐MnO_2_, including {110}, {101}, {100}, and {001}, and their proton adsorption have been studied recently by DFT calculations (**Figure** **5**a).^48^ Their surface energies increased in the order of {110}, {101}, {100}, and {001} (Figure 5a). Facets with presence of higher number of oxygen atoms per unit area offer higher number of adsorption sites, resulting in higher adsorption pseudocapacitance. {001} facet displayed the highest calculated adsorption pseudocapacitance of 1.61 mF cm^−2^, followed by {100}, {101}, and {110} facets. The adsorption pseudocapacitance values of different facets increased proportionally according to their surface energies. Naturally, the lowest surface energy facet is the preferential grown surface; therefore, surface engineering routes to optimize the growth of high‐energy facets are essential to increase pseudocapacitance values. Moreover, energy barriers for proton diffusion into near surface and into the bulk of β‐MnO_2_ are much less in {001} facet compared to {110} facets (Figure 5b). The proton diffusion coefficient into near surface of {001} is 35 orders of magnitude higher than that of {110} facets, favoring the proton diffusion along {001} direction. 1D tunnel of β‐MnO_2_ vertical to {001} facets could facilitate the proton diffusion, resulting in high diffusion coefficients. Thus, optimizing tunnel orientation via facet engineering is also expected to improve the charge storage performance of the electrode materials. Experimental work addressing facet engineering has been reported. For example, Co_3_O_4_ nanocrystals with predominant high‐energy exposed facets {110}, as shown in transmission electron microscopy (TEM) results (Figure 5c), were prepared via hydrothermal thermal synthesis, varying precursor concentrations, followed by thermal calcinations at 250 °C.^49^ Nanorods, nanobelts, and nanosheets with high‐energy {110} exposed facets delivered higher specific capacity value compared to nanocubes or nanooctahedra with low‐energy (100) or (111) exposed facets (176.8 F g^−1^ at 1 A g^−1^ vs 20 F g^−1^ at 5 mV s^−1^). Cuboctahedral NiO mesocrystals, with increased high‐energy {001} exposed facets, were prepared by solvothermal routes and displayed a specific capacity of 1039 F g^−1^ at 1 A g^−1^, which was higher for the NiO mesocrystals with lower‐energy {111} exposed facets.^50^ α‐Fe_2_O_3_ with tunable exposed facets was prepared by a solvent‐mediated precipitation route with varied ethylene glycol concentration.^51^ NiCo_2_O_4_ microspheres surrounded by nanowires, containing different exposed reactive planes—(001) and $\left(01\bar{1}\right)$, were prepared by the hydrothermal method and post‐thermal calcinations.^52^ They displayed specific capacity of 1284 and 986 F g^−1^ at 2 and 20 A g^−1^, respectively, and long‐term stability with only 2.5% capacity loss after 3000 cycles. The *c*‐axis preferentially oriented TiO_2_ nanotubes, grown by annealing amorphous TiO_2_ nanotubes in vacuum, showed increased capacity compared to randomly grown TiO_2_ nanotubes (8.21 mF cm^−2^ vs 0.32 mF cm^−2^ at 100 mV s^−1^).^53^ Note that the high number of oxygen vacancies in TiO_2_ influenced the electrochemical response; this defect chemistry will be discussed in the following section.

Facet engineering of metal hydroxides for supercapacitors has been scarcely reported, to the best of our knowledge; nevertheless, controlling the preferential growth of hydroxides with highly active facets would vary electrochemical activity, changing bulk ion diffusion coefficients and the corresponding electrode performance.

Despite some results that have been reported for aqueous supercapacitor electrodes, facet engineering is still very little studied in the field. Further studies, looking into the charge storage ability of different facets and understanding and optimization of highly active facets, are crucial to advance knowledge and to develop metal oxide and hydroxide–based materials for aqueous supercapacitors.

#### 3.1.2 Defect Engineering

Defect engineering is an effective route to modify the properties of materials as their physicochemical response changes with the formation of structural or chemical imperfections. Defects may change materials' electronic structure, such as band structure, and form defect states in the bandgap, either as donor or as acceptor, which vary carriers' concentration, chemical potential, and work function, affecting directly the electron conductivity and the redox activity of the materials. Surface defects change atomic configuration and electronic states at the surface, and vary the surface activity of the materials. Intrinsic defect engineering, via formation of nonstoichiometric metal oxides or hydroxides, such as oxygen or cation vacancies, without addition of other elements (extrinsic defects), can significantly alter the physiochemical properties of materials and sum up the advantages of low‐cost material modification routes. This approach has been progressively employed to enhance the supercapacitive performance of metal oxide and hydroxide materials.

**Figure 6:**
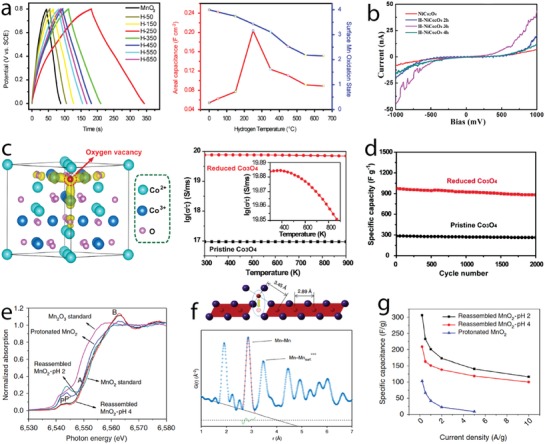
a) Left: charge–discharge curves at 1 mA cm^−2^; right: capacitance values and Mn valence states of MnO_2−_ *_x_* electrodes prepared by hydrogen reduction at different temperatures. b) Current–voltage plot of NiCo_2_O_4−_ *_x_* nanowires reduced with different hydrogen treatment times. c) Left: crystal structure of Co_3_O_4_ containing oxygen vacancy; right: electron conductivity of Co_3_O_4−_ *_x_* and Co_3_O_4_ at different temperatures (calculated by DFT method); and d) specific capacity values of Co_3_O_4−_ *_x_* and Co_3_O_4_ during charge–discharge cycling. e) XANES spectra of H*_x_*MnO_2_ and MnO_2_ reassembled at different pH values; f) top: illustration of Mn surface Frenkel; bottom: in‐plane Mn–Mn distance and Mn–Mn_surf_ revealed by pair distribution function analysis of MnO_2_ reassembled at pH 2; g) specific capacitance values at different specific currents of H*_x_*MnO_2_ and MnO_2_ reassembled at different pH values. a) Reproduced with permission.[[qv: 34a]] Copyright 2014, Elsevier Ltd. b) Reproduced with permission.[[qv: 34b]] Copyright 2016, Royal Society of Chemistry. c,d) Reproduced with permission.[[qv: 58a]] Copyright 2014, John Wiley and Sons. e–g) Reproduced with permission.^61^ Copyright 2017, Springer Nature.

MnO_2_ containing controllable oxygen vacancies (MnO_2−_ *_x_*) was prepared by thermal hydrogen reduction at different temperatures.[[qv: 34a]] These defects enhanced the pseudocapacitive response in MnO_2−_ *_x_*, leading to maximized areal capacitance to 0.2 F cm^−2^ at 1 mA cm^−2^ in 0.5 m Na_2_SO_4_ as compared to 0.05 F cm^−2^ at 10 mV s^−1^ of the stoichiometric MnO_2_. The oxygen vacancies induced the formation of mixed valence Mn (+4, +3, and +2) for charge balancing and reduced the charge transfer resistance that is assigned to enhanced capacity. However, excess of oxygen vacancies decreased capacity due to increased charge transfer resistance, which highlights how important is to control this defect (**Figure** **6**a). First principles DFT calculation on oxygen vacancy formation at β‐MnO_2_ grain boundary and single‐layered MnO_2_ revealed the existence of spin‐polarized gap states and metallic behavior at the grain boundary and half‐metallic behavior of the single layer, which could also be associated with the increased capacity of the MnO_2−_ *_x_* electrode.^54^

Oxygen‐deficient α‐Fe_2_O_3_ nanorods were prepared by thermal decomposition of hydrothermally grown FeOOH in N_2_ atmosphere and evidenced improved donor density.^55^ α‐Fe_2_O_3_ displays n‐type semiconductive behavior and its donor densities increased from 5.5 × 10^18^ to 8.1 × 10^19^ cm^−3^ after oxygen vacancy formation. As a result, the equivalent series resistance and charge transfer resistance of the electrode decreased. The creation of oxygen vacancies in Fe_2_O_3_ nanorods with high mass loading of 4.3 mg cm^−2^ led to increased pseudocapacitance values of 382.7 mF cm^−2^ at 0.5 mA cm^−2^ in 3 m KCl electrolyte and increased stability with 95.2% capacitance retention after 10 000 cycles.

Oxygen vacancies were introduced in VO_2_ by thermal reduction in H_2_ atmosphere and increased the material conductivity by almost three orders of magnitude and created essentially low V vacancies.^56^ Those intrinsically enhanced properties improved the pseudocapacitive performance of the electrode in 1 m Na_2_SO_4_ aqueous electrolyte, which displayed specific capacitance of 300 F g^−1^ (for a symmetric cell, the capacitance of the single electrode was not reported) compared to 76 F g^−1^ of stoichiometric VO_2_ and good cycling stability with 96% capacitance retention after 500 charge–discharge cycles (which generally quickly dropped for VO*_x_*‐based electrode working in aqueous electrolytes).

Controllable oxygen‐deficient spinel NiCo_2_O_4_ electrodes were prepared by thermal reduction in H_2_ atmosphere with different treatment times.[[qv: 34b]] Electron conductivity increased for treatment up to 3 h and decreased when the H_2_ treatment time was extended for 4 h as revealed by in situ electrical scanning tunneling microscopy–TEM (Figure 6b). Formation of optimal oxygen vacancies and a structural disorder surface enhanced redox response of the nonstoichiometric NiCo_2_O_4_ electrode, which delivered a high capacity of 2.13 F cm^−2^ at 1 mA cm^−2^ (which is 240% increment as compared to the stoichiometric spinel oxide). Oxygen vacancies formed by H_2_ reduction on MnMoO_4_ also improved redox response.^57^ Oxygen vacancies were also produced by chemical reduction of electrodes with NaBH_4_ (a reducing agent).^58^ For example, conductivity of oxygen‐deficient Co_3_O_4_ electrodes increased as revealed by DFT calculation (Figure 6c), which resulted in enhanced storage capacity (978 F g^−1^) (Figure 6d).

Monolayer NiTi‐layered double hydroxide (LDH) was grown by in situ reverse microemulsion technique.^59^ Ni^3+^ surface state in the monolayer increased compared to the bulk materials, which was probably related to the oxygen vacancies. DFT calculations revealed the transformation from semiconducting stoichiometric NiTi hydroxide to gapless half‐metallic Ni^3+^‐associated NiTi hydroxide. This property resulted in an electrode delivering high specific capacity, 2310 F g^−1^ at 1.5 A g^−1^, and 82% capacity retention after 3000 charge–discharge cycles.

Oxygen vacancy engineering has shown to be an efficient route to tailor the charge storage performance of metal oxide electrodes. Oxygen vacancies on electrode materials are currently produced via two main routes: hydrogen treatment of the stoichiometric materials at elevated temperature and thermal annealing of metal hydroxides in inert atmosphere. Other engineering routes to produce oxygen vacancies, such as high‐energy particle bombardment and forming oxygen vacancies during materials' growth processes, can also be interesting routes to tailor electrode performance.

While several works addressing oxygen vacancy engineering have been reported for aqueous supercapacitors, metal vacancy engineering is much less studied. Among those, Ni^2+^ vacancies in NiO induced the formation of Ni^3+^ and enhanced the storage capacity.^60^ Recently, the effect of cation point defects on the pseudocapacitive response of δ‐MnO_2_ electrodes was studied in detail.^61^ In this work, controllable Mn point defects and Mn^3+^/Mn^4+^ ratios in δ‐MnO_2_ were implemented by exfoliation and reassembling of crystalline δ‐H*_x_*MnO_2_ nanosheets at pH of 2 or 4. Mn^3+^ content increased in the reassembled δ‐MnO_2_ compared to the crystalline δ‐H*_x_*MnO_2_; the lowest pH value increased the reduction of Mn^4+^ to Mn^3+^, which increased Mn^3+^ content. The average Mn valence states calculated from XANES were 3.59, 3.36, and 3.24 for H*_x_*MnO_2_, and δ‐MnO_2_ assembled at pH 4 and pH 2, respectively (Figure 6e). High‐energy X‐ray scattering and pair distribution function analysis revealed the presence and the increase in surface Frenkel Mn defects (Figure 6f–g) in the assembled δ‐MnO_2_ and the Frenkel defect concentration increased 30%, when lowering the pH from 4 to 2, since the in‐plane Mn was expelled preferable in higher acidic condition to form Mn vacancies. Assembled δ‐MnO_2_ nanosheets favored preferentially vacancy formation and the reduction of Mn^4+^ rather than the crystalline H*_x_*MnO_2_, due to the steric hindrance by H*_x_*MnO_2_ interlayer that consisted of protons and water. The increased Mn surface Frenkel defects in assembled δ‐MnO_2_ at low pH of 2 enhanced the pseudocapacitive response. The specific capacitance values were 306, 209, and 103 F g^−1^ at 0.2 A g^−1^ for δ‐MnO_2_ assembled at pH 2 and pH 4, and crystalline H*_x_*MnO_2_ (Figure 6g). Enhanced pseudocapacitance related to the formation of Frenkel defects could be associated with easier intercalation reaction, and the increased conductivity with the presence of more defect‐induced Mn^+3^ content. Although this work suggested that cation vacancy engineering would enlarge the storage capacity, the cycle life of the assembled δ‐MnO_2_ is still an important drawback that imposes further research.

#### 3.1.3 Functional Architecture Engineering

Architecture engineering is currently a very dynamic research stream toward optimal charge storage performance of aqueous supercapacitors. The architecture of the materials composing the electrode affects specific surface area and electrolyte diffusion ability, which in turn control the number of surface active sites for the charge storage processes, thereby governing storage capacity as revealed by Equation (11). A wide array of nanostructured metal oxides and hydroxides with different dimensionality have been designed. Low dimension lengths generally contribute to increased specific surface area and reduced diffusion length for bulk reactions. 0D nanostructures such as nanoparticles, 1D nanostructures such as nanowires, nanorods, and nanotubes, and 2D nanostructures such as layered nanosheets and nanoplatelets have been studied and in general evidence good charge storage performance. These nanostructures can be grown directly onto current collectors, forming 3D nanostructured electrode architectures containing interlinked nanostructures, which prevent agglomeration effects, accommodate volume expansion during Faradaic redox reactions, and reduce the contact resistance since there is no need of adding binders. The design of architectures based on nanostructured morphologies, accounting for optimal weight and volume distribution of the active material, has been proposed for enhancing supercapacitor performance. Architectures assembling different nanostructures are likely to provide more active sites for redox reactions. Hollow structures containing several shells with optimized empty spacing can enhance the volumetric capacity and can minimize stress induced by contraction/expansion of the material. Thus, tuning the design and production of electrode architectures based on nanostructured materials and the comprehension of their growing processes is an important route to tailor supercapacitor electrodes toward enhanced charge storage performance.

**Figure 7:**
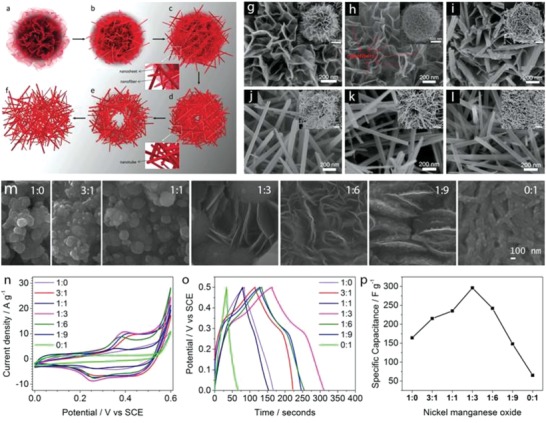
a–f) Schematic views and g–l) field‐emission gun (FEG)‐SEM images of nanostructured MnO_2_ growth with hydrothermal time: a,g) 1 h, b,h) 1.5 h, c,i) 2 h, d,j) 4 h, e,k) 8 h, and f,l) 12 h. m) FEG‐SEM images of the Ni–Mn oxide films electrodeposited at different Ni:Mn ratios of 1:0, 3:1, 1:1, 1:3, 1:6, 1:9, and 0:1; a scale bar at the bottom is applied for all images in the figure. n) Cyclic voltammograms at the scan rate of 20 mV s^−1^, o) charge–discharge curves at the specific current of 1 A g^−1^, and p) the specific capacity values of the films with different Ni:Mn ratios. a–l) Reproduced with permission.^62^ Copyright 2014, John Wiley and Sons. b) Reproduced with permission.^63^ Copyright 2015, Royal Society of Chemistry.

Different MnO_2_ nanostructures were prepared by hydrothermal routes with varied reaction times.^62^ This route produced an active material composed of spherical microparticles containing different nanostructures, which changed morphology when increasing the reaction time, leading to the formation of nanosheets, nanofibers, and nanotubes. Urchin spheres, consisting of nanotubes and randomly distributed nanotubes, were formed when the reaction time was set to 8 and 12 h. δ‐MnO_2_ was formed under reaction times lower than 2 h, while α‐MnO_2_ was formed under longer reaction time. The MnO_2_ nanostructure growing process is schematically depicted in **Figure** **7**a–f. The growth of microspheres involves nanosheets associated with the formation of layered δ‐MnO_2_ phase, which favored the growth of 2D features (Figure 7a,g). The δ‐MnO_2_ nanosheets were metastable states, resulting in diffusion of δ‐MnO_2_ domains into α‐MnO_2_ nanofiber nuclei (Figure 7b,h) and anisotropic growth of the nanofiber with increased reaction time (Figure 7c,i) until complete transformation of the nanosheets (Figure 7d,j). This process was due to the high thermodynamic stability of the 2 × 2 tunnel α‐MnO_2_ compared to the layered δ‐MnO_2_. The surface of α‐MnO_2_ nanofibers in contact with the reaction media was under Ostwald ripening process (recrystallization process), which was faster than that in the inner side, leading to the formation of hollow nanotubes upon complete growth of the nanofibers. Ostwald ripening continued with the increased reaction time, resulting in complete transformation of the nanofibers into nanotubes (Figure 7e,k), as revealed by 3D electron tomography. Nanotubes with open large hollows formed and dispersed in solution rather than in the urchin sphere with longer hydrothermal time due to the influence of acid etching (Figure 7f,l).

Double Ni–Mn oxides were prepared by cathodic electrodeposition on stainless steel followed by thermal annealing for the supercapacitor electrodes^63^ and different morphological changes were observed when varying Ni:Mn ratios (Figure 7m). A novel surface morphology, with a complex nanostructure composed of the nanosheets linked with the texture particles, was formed at Ni:Mn of 1:3. The morphological changes when varying Ni:Mn ratios were assigned to the differential growth of the unitary metal oxides. Cyclic voltammograms of these oxide films evidenced redox peaks shifted to lower potentials when the Mn content increased (Figure 7n). The redox response increased and then decreased when the Mn content in the binary Ni–Mn oxides gradually increased, reaching the maximum response at Ni:Mn of 1:3. This response is a consequence of a synergistic effect of the redox reactions and the architecture of the Ni–Mn oxides. The specific capacity values (Figure 7p) calculated from the galvanostatic charge–discharge at the specific current of 1 A g^−1^ (Figure 7o) evidenced maximum values of 300 F g^−1^ in the Ni–Mn oxide film with Ni:Mn of 1:3 in agreement with the synergistic redox response observed in the cyclic voltammograms and with the enhanced specific surface area provided by the new electrode architecture. This electrode also displayed very good behavior under continuous charge–discharge cycling, with a capacity retention of nearly 100% after 1500 cycles.

The morphology of Ni(OH)_2_ films prepared by hydrothermal routes was tailored under different reaction temperatures, which can control nucleation and growing kinetics.^64^ Different morphologies were obtained, including nanoflakes, stacked nanoflakes, nanobelts, and nanoribbons, which were formed when the reaction temperature increased, displaying surface areas of 29.04, 16.98, 15.34, and 13.29 m^2^ g^−1^, respectively.

**Figure 8:**
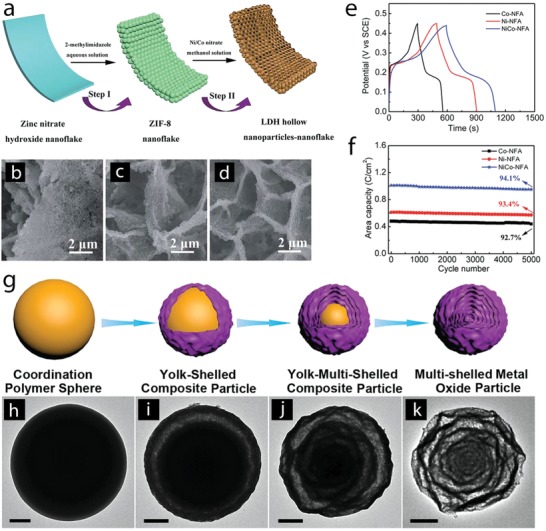
a) Schematic illustration depicting the formation of the hollow NFAs; FEG‐SEM images of b) Co, c) Ni, and d) Ni–Co NFAs; and e) charge–discharge curves at 4 mA cm^−2^ and f) cycling curves for 5000 charge–discharge cycles of NFAs. g) A schematic illustration of the formation of a multishelled metal oxide particle from a coordination polymer; h) TEM images of Ni–Co coordination polymer and the morphological changes after calcination at i) 360 °C, j) 390 °C, and k) 500 °C. Scale bars in TEM images are 200 nm. a–f) Reproduced with permission.^66^ Copyright 2016, Royal Society of Chemistry. g) Reproduced with permission.^67^ Copyright 2017, John Wiley and Sons. h–k) Reproduced with permission.^68^ Copyright 2017, John Wiley and Sons.

Metal oxides and hydroxides transformed from metal organic frameworks (MOFs) are currently emerging as an attractive route to design new architectures for supercapacitor electrodes. MOFs possess high surface area and high porosity due to their structural order, which consists of different metal centers and ligands. Thus, structural transformations of MOFs to oxide and hydroxide phases, while retaining high specific surface area and porosity, are expected to work as high‐performance electrode materials.^65^ For example, Ni*_x_*Co_3−_ *_x_*O_4_ nanoparticles were prepared by thermal annealing of Ni–Co–MOF‐74 nanocrystals at 400 °C, displaying a spindle‐like morphology preserved from Ni–Co–MOF‐74 with high surface area of 64–117 m^2^ g^−1^.[[qv: 65b]] The obtained electrodes presented a maximum specific capacity of 797 F g^−1^ at 1 A g^−1^ and good long‐term stability. Co, Ni, and Ni–Co hydroxide hollow nanoparticle–nanoflake architectures (NFAs) on Ni foam were prepared by immersing a ZIP‐8 nanoflake array onto Ni foams (prepared by transformation of zinc nitrate hydroxide nanoflake array in 2‐methylimidazole solution) in nickel nitrate and cobalt nitrate dissolved methanol solution (**Figure** **8**a).^66^ During the transformation reaction, breaking of ZIP‐8 coordination bonds and oxidation of Co^2+^/Ni^2+^ to Co^3+^/Ni^3+^ occurred, and Zn^2+^ ions coprecipitated with Ni^2+^/Co^2+^ and Ni^3+^/Co^3+^ ions due to Kirkendall effect, resulting in the formation of the hollow morphology (Figure 8b–d). This novel Ni–Co hydroxide architecture delivered a capacity of 2.04 C cm^−2^ (971.4 C g^−1^) at 4 mA cm^−2^ (1.9 A g^−1^) and 0.84 C cm^−2^ (400 C g^−1^) at 48 mA cm^−2^ (22.9 A g^−1^) and a capacity loss of 5.9% after 5000 cycles (Figure 8e,f).

Metal oxides and hydroxides with more complex nanostructured architectures are also being developed for supercapacitor electrodes such as core–shell, yolk–shell, and multishell structures.^67^ Multishelled particles of Mn–Co oxide, Mn–Ni oxide, Zn–Mn oxide, and Mn–Co–Ni oxide were synthesized by thermal treatment of the corresponding amorphous coordination polymer precursor spheres.^68^ Amorphous coordination polymers can be incorporated with most of the transition metal cations, thereby allowing tunable composition of their derived compounds. The thermal oxidation of those compounds resulted in the formation of seven‐layered multishelled particles evidenced by TEM (Figure 8g–k). This process is related to the oxide spallation phenomena during oxidation, in which the particle shrinkage, due to weight loss, created residual stress in the oxide layer. When the oxide layer reached a critical thickness, the spallation occurred and a yolk–shelled structure was formed at 360 °C (Figure 8i). The oxide layer displayed crumpled morphologies as a result of the residual stress during phase transformation. The contraction continuously occurred with heating, forming yolk–multishelled particles at 390 °C (Figure 8j) and seven‐layered multishelled particles at 500 °C (Figure 8k). The Ni–Co oxide multishelled particles delivered a maximum specific capacity of 1908 F g^−1^ at 2 A g^−1^ and excellent cycling stability with 93.6% capacity retention after 20 000 cycles. In situ electrochemical TEM results revealed that the multishelled particles provided good volume change accommodation.

Many routes, such as hydrothermal, electrodeposition, and sol–gel, or more recent ones such as MOF conversion, have been used to fabricate novel nanoarchitectures for high‐performance aqueous supercapacitors. By controlling the synthesis parameters, it has been possible to control material growth and to create novel morphologies assembled in different architectures. However, often nanoarchitectures may evolve under an unpredictable way. Advanced electrode design techniques, which can accurately control the growth of the intended nanostructures, can advance significantly the development of novel electrode architectures, not only for aqueous supercapacitors but also for many other applications. Presently, 3D printing techniques are evolving very fast and some interesting achievements have been reported for 3D printing with graphene‐based electrodes.^69^ Despite important advances, the use of this technique to design metal compound electrodes is still at early infancy, to the best of authors' knowledge. The most important factor for current 3D printing technologies is the printable ink. Some results on hydroxide ink were recently reported and eventually these will nucleate the rising of 3D printing techniques to design metal oxide and hydroxide electrodes, for example.^70^

### 3.2 Extrinsic Materials Engineering and Functionalization

#### 3.2.1 Doping

Doping is widely used to engineer materials' properties by adding, intentionally, a small quantity of extrinsic atoms. Doping intends to control relevant properties such as electronic structure, charge carrier concentration, and phases, which greatly influence electrical conductivity, chemical potential, and surface activity. As these properties are associated with the redox activity of electrode materials and govern their charge storage performance, relevant work on either metal or nonmetal doping of metal oxide and hydroxide electrodes has been reported.

**Figure 9:**
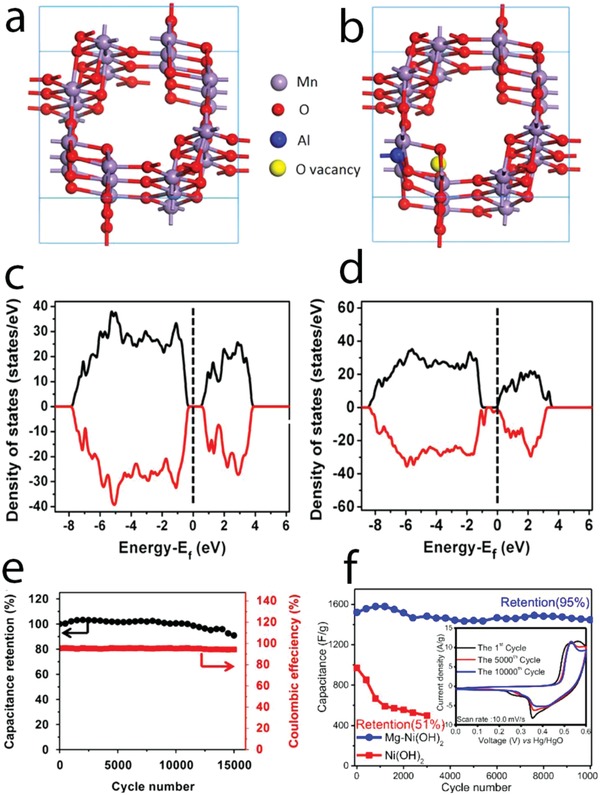
Crystal structures of a) MnO_2_ and b) Al‐doped MnO_2_; density of states of c) MnO_2_ and d) Al‐doped MnO_2_; e) charge–discharge cycling ability and Coulombic efficiency of Al‐doped MnO_2_ at specific current of 2 A g^−1^ for 15 000 cycles. f) Capacity retention of Ni(OH)_2_ for 3000 charge–discharge cycles and Mg‐doped Ni(OH)_2_ for 10 000 charge–discharge cycles at 10 A g^−1^; inset shows cyclic voltammograms of Mg‐doped Ni(OH)_2_ after 1st, 5000th, and 10 000th cycles. a–e) Reproduced with permission.^72^ Copyright 2014, Elsevier Ltd. g) Reproduced with permission.^76^ Copyright 2016, American Chemical Society.

Ce^3+^‐doped MnO_2_ was prepared by the hydrothermal route.^71^ Ce^3+^ doping induced phase transformation from β‐MnO_2_ to α‐MnO_2_ with increased tunnel size due to the stabilization of 2 × 2 tunnels by Ce^3+^. Optimal doping concentration (5.6% of Ce to Mn) produced finer nanorods, displaying decreased diameter and length due to inhibition of growth along and perpendicular to the [001] direction, with presence of Ce^2+^ in 2 × 2 tunnel that increased material electrical conductivity. Thus, Ce^3+^ doping increased charge storage capacity by one order of magnitude compared to undoped MnO_2_. Al‐doped α‐MnO_2_ materials (**Figure** **9**a,b) with different dopant concentrations suffered morphological changes based on microspheres with different nanostructures such as nanoneedles, nanoparticles, and fine nanosheets.^72^ Densities of states calculated by DFT revealed an increase of the Fermi level energy and doping states near the valence and conduction bands, narrowing the bandgap and increasing conductivity (Figure 9c,d). Al‐doped α‐MnO_2_ microspheres (1.75 wt%) delivered specific capacitance values of 213 and 146 F cm^−3^ in 0.5 m Na_2_SO_4_ at 0.1 A g^−1^ and at mass loading of ≈4 mg cm^−2^, good Coulombic efficiency (nearly 100%), and good cycle life, maintaining 91% of the initial capacitance after 15 000 cycles (Figure 9e). Similarly, Fe acted as electron donor in Fe‐doped Co_3_O_4_ to increase the density of states near the Fermi level and the conductivity, thereby enhancing charge storage performance.^73^ Fe‐doped MnO_2_ induced significant morphological changes and a phase transformation, from mixed α‐MnO_2_ and γ‐MnO_2_ to mixed α‐MnO_2_, γ‐MnO_2_, and ε‐MnO_2_ phases.^74^ Optimal Fe dopant concentration reduced both equivalent series resistance and charge transfer resistance, as evidenced by electrochemical impedance spectroscopy, thus enhancing the pseudocapacitive performance. Fe‐doped mixed α‐, γ‐, and ε‐MnO_2_ with high mass loading of 5 mg cm^−2^ delivered specific capacitances of 267.0 and 183.1 F g^−1^ at 0.1 and 5.0 A g^−1^, respectively, with 3.2% capacitance loss after 2000 charge–discharge cycles. Mn_3_O_4_ octahedron nanocrystals were doped with different transition metal ions, including Cr, Co, Ni, and Cu.^75^ Divalent cations (Co, Ni, and Cu) occupied tetrahedral sites of Mn_3_O_4_ and a trivalent cation (Cr) occupied octahedral sites of Mn_3_O_4_. Doping with Co and Cu decreased the average rhombic length of the octahedron, whereas doping with Cr and Ni slightly increased these lengths. Cr‐doped Mn_3_O_4_ delivered enhanced specific capacitance value compared to the undoped one (272 F g^−1^ vs 202 F g^−1^ at 0.5 A g^−1^). Contrarily, Co‐doped Mn_3_O_4_ displayed similar capacity and Ni‐ and Cu‐doped Mn_3_O_4_ decreased the capacity. The role of different transition metal doping on the charge storage mechanism is still unclear and no concise mechanism has been established, but existing results highlight the importance of doping elements. Further research is thus necessary to establish the mechanism that governs the doping effects on the charge storage capacity.

α‐Ni(OH)_2_ doped with Mg (1Mg to 13Ni) was prepared by in situ ion‐exchange reactions using Mg(OH)_2_ as sacrificial template and dopant.^76^ The spontaneous ion‐exchange reaction, due to differences in the solubility product of Mg(OH)_2_ and Ni(OH)_2_, led to simultaneous formation of Ni(OH)_2_ and doping with Mg. Mg‐doped α‐Ni(OH)_2_ displayed high Brunauer–Emmett–Teller surface area (202 m^2^ g^−1^) and pore volume of 0.56 cm^3^ g^−1^, which resulted in high specific capacity values: 1931 and 1496 F g^−1^ at 0.5 to 20 A g^−1^ in 6 m KOH, respectively. The capacity retained 95% of its initial value after 10 000 cycles, which is an interesting achievement considering the poor stability of Ni(OH)_2_‐based electrodes (Figure 9f). Morphological stableness was observed by scanning electron microscopy (SEM), thanks to the incorporation of Mg(OH)_2_ into the Ni(OH)_2_ structure that also stabilized its redox reactions.

S‐doped V_6_O_13−_ *_x_* electrodes were prepared by thermal annealing of V_3_O_7_ nanowires in nitrogen atmosphere containing sulfur vapor.^77^ S doping increased electron conductivity and ion diffusion, resulting in high gravimetric capacitances, 1350 F g^−1^ (0.72 F cm^−2^) at 1.9 A g^−1^. Nevertheless, the specific capacitance decreased quickly after 200 cycles to 47.7% of the initial capacitance due to chemical dissolution of V^3+^ during charge–discharge cycling. Although a carbon coating is required to stabilize the electrode, the high capacitance obtained by S doping suggested that nonmetal doping could be employed to enhance charge storage. α‐Ni(OH)_2_ nanowires doped with S^2−^ in their interlayer gallery were produced by an anion‐exchange reaction between intercalated SO_4_^2−^ and S^2−^.^78^ This exchange reaction reduced the crystallinity and created nanocavities distributed over the nanowires since the radius of SO_4_^2−^ is larger than S^2−^, forming mesoporous nanowires. The increase of S^2−^ content resulted in decreased surface area and larger pore diameter related to the development of mesopores. The optimal S^2−^‐doped α‐Ni(OH)_2_ showed a high capacity value of 2223 F g^−1^ at 1 A g^−1^ (compared to 376 F g^−1^ of undoped ones). β‐FeOOH doped with 5.06% of F was prepared via a hydrothermal route^79^ and the material displayed low resistivity, with an area normalized electrical resistance of 2.211 Ω cm^−2^ measured by the four‐probe method. The reduction of the bandgap in β‐FeOOH from 1.05 to 0.2 eV in F‐doped β‐FeOOH, as calculated by DFT, resulted in metal‐like property significantly enhancing conduction. Thus, F‐doped β‐FeOOH enlarged the pseudocapacitive response (1.12 F cm^−2^ at 1.0 mA cm^−2^), which is higher than that of F‐free β‐FeOOH (0.63 F cm^−2^ at 1.0 mA cm^−2^) and the specific surface area was also reduced after F doping (51.896 m^2^ g^−1^ vs 63.099 m^2^ g^−1^). This material also showed excellent response with 83.0% capacitance retention under high current of 100 mA cm^−2^ and good capacitance retention after 5000 cycles. α‐(Ni/Co)(OH)_2_ doped with metaborate (BO_2_^−^) in their interlayers showed strong bonds with the hydroxide layers (other ions that can be intercalated such as nitrate, chloride, and sulfate showed weak bonding with the hydroxide layers), stabilized the hydroxide structure, and improved the material charge storage ability.^80^ However, a composite formed with graphene was still necessary to increase the material conductivity, an issue that will be discussed in the following section.

#### 3.2.2 Compositing and Hybridizing

Metal oxides and hydroxides are wide‐bandgap materials with low electron conductivity. This property leads to the decrease of the redox‐active sites due to the inefficient electron transport to the electrode/electrolyte interface. Thus, the development of hybrid or composite materials composed of redox‐active compounds dispersed over conducting materials has been an attractive route to improve the electrode charge storage performance. Metals or metal alloys such as Ni, Ni–Cu, and Au and carbon‐based materials, such as graphene and carbon nanotubes, have been employed for fabricating composites with metal oxides and hydroxides. While composites with metals mainly aim at increasing conductivity, composites with carbon can provide additional charge storage due to the contribution of the double layer, increasing storage capacity and response rate. Agglomeration and lack of chemical stability of the redox materials could be minimized by forming composites with more stable redox activity. Moreover, composites may display different advantageous architectures that facilitate diffusion of electrolyte ions.

**Figure 10:**
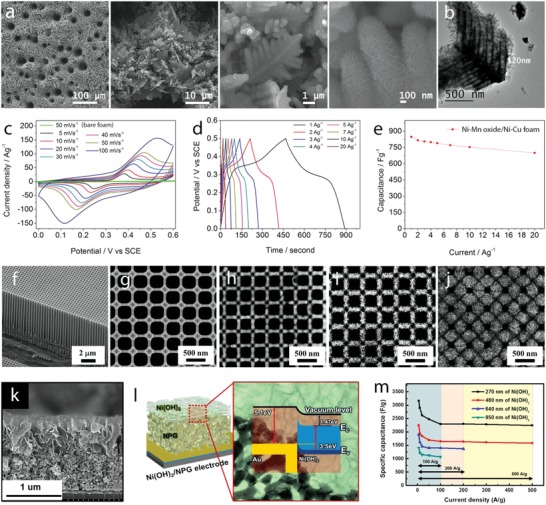
a) FEG‐SEM images of Ni–Mn oxide layer deposited on Ni–Cu foam at different magnifications; b) TEM image of Ni–Mn oxide covered Ni–Cu dendrite; c) cyclic voltammograms at different scan rates, d) charge–discharge curves, and e) capacity values at different currents of the Ni–Mn oxide/Ni–Cu foam electrode. f) Cross‐sectional and g) top‐view FEG‐SEM images of Ni nanopore arrays; top‐view FEG‐SEM image of Ni nanopore arrays covered with MnO_2_ with mass loadings of h) 80 µg cm^−2^, i) 240 µg cm^−2^, and j) 400 µg cm^−2^. k) Cross‐sectional FEG‐SEM image and l) schematic illustration of Ni(OH)_2_/nanoporous Au electrode and band alignment of Au and Ni(OH)_2_; m) capacity values at different specific currents of Ni(OH)_2_/nanoporous Au with different thicknesses of Ni(OH)_2_ layer. a–e) Reproduced with permission.^81^ Copyright 2015, Royal Society of Chemistry. f–j) Reproduced with permission.^85^ Copyright 2014, John Wiley and Sons. k–m) Reproduced with permission.^89^ Copyright 2016, Elsevier Ltd.

Novel Ni–Mn oxide/Ni–Cu foam electrodes were deposited on stainless collectors by a two‐step electrodeposition route: first Ni–Cu foam and then Ni–Mn oxide. These electrodes utilized the redox response of Ni–Mn oxide for charge storage and the high electronic conductivity of the Ni–Cu foam.^81^ The Ni–Cu foams presented an open porous 3D morphology with randomly distributed micrometric pore size. The pore walls were constituted of randomly interconnected dendrites forming an open porous 3D percolation network, thus creating a good electron conducting pathway and favoring the diffusion of the electrolytes. Ni–Mn oxide uniformly covered the Ni–Cu dendrites of the foams and displayed percolating nanosheet‐like morphologies with porous structure (**Figure** **10**a,b). The increase of the volume fraction of Ni–Mn oxide films obtained by electrodeposition over thicker foams increased specific capacitance of the electrode. Thanks to this architecture, the electrode delivered good redox activity and maximal specific capacity of 848 F g^−1^ at 1 A g^−1^ (Figure 10c,d). Interestingly, these electrodes displayed excellent response rate with capacity retention of 83% when the specific current increased from 1 to 20 A g^−1^ (Figure 10e). These promising results were the result of the hierarchical architecture consisting of double 3D percolation networks of Ni–Mn oxide nanosheets and Ni–Cu dendrites that enhanced the interfacial area of the active material, facilitating the accessibility of ions and charge transfer.

Other metal oxide/hydroxide–metal composites/hybrids have also been reported such as Fe_2_O_3_–Ni nanotubes,^82^ RuO_2_–Au foam,^83^ MnO_2_–Ni nanowires,^84^ MnO_2_–Ni nanopore arrays,^85^ Ni–Mn oxyhydroxide–nanoporous Ni–Mn alloy,^86^ and Ni–Co oxyhydroxide on Ni–Co dendrites, all displaying enhanced electrochemical response as charge storage materials.^87^

α‐Fe_2_O_3_ nanoneedles and MnO_2_ nanosheets were grown on ultrafine Ni nanotube arrays.^82^ Ni layer on a ZnO nanorod array was first formed, followed by anodic deposition of FeOOH or MnO_2_. The local pH, near the electrode, decreased during electrodeposition of FeOOH and dissolved ZnO; the post‐thermal treatment transformed FeOOH into α‐Fe_2_O_3_, leading to formation of α‐Fe_2_O_3_ nanoneedles–Ni nanotubes. HCl was used to etch the ZnO nanowires in MnO_2_–Ni–ZnO and to form MnO_2_ nanosheets–Ni nanotubes. The efficient electron transport of Ni nanotubes, together with the formation of conformably nanostructured active oxide coatings, provided good charge storage performance. α‐Fe_2_O_3_–Ni and MnO_2_–Ni nanotubes delivered capacitance values of 418.7 F g^−1^ at 10 mV s^−1^ and 440.7 F g^−1^ at 5 mV s^−1^, respectively, with 7–8% capacitance loss after 5000 charge–discharge cycles. RuO_2_–porous Au electrodes formed via the growth of porous foam‐like Au layers followed by the growth of a hydrous RuO_2_ coating.^83^ This electrode delivered areal capacitance of 3.25 F cm^−2^ at 0.1 mV s^−1^, which is comparable to the state of the art in Li microbatteries. MnO_2_ was grown by magnetic field–driven selective deposition on ultralong Ni nanowires, with length up to 1 mm, followed by anodic electrodeposition of MnO_2_.^84^ The ultralong nanowires provided high surface sites to support high mass loading of MnO_2_ on the current collector and maintained thin layers of MnO_2_ on nanowires. The electrode, with MnO_2_ mass loading of 3.51 mg cm^−2^, presented gravimetric and areal capacitances of 214 F g^−1^ and 750 mF cm^−2^ at 1 mV s^−1^, respectively, and stability under charge–discharge cycling with no capacitance loss after 20 000 cycles. Ni rectangular nanopore arrays, prepared by a replication route, from an anodic aluminum oxide membrane template, via alternative deposition and etching (Figure 10f,g), were used to support the growth of MnO_2_ growth.^85^ MnO_2_ coating could maintain the pores of the Ni arrays, whose size decreased with increasing MnO_2_ loading (Figure 10h–j), thus promoting diffusion of electrolyte ions and enhancing the response rate. As a result, the MnO_2_–Ni nanopore array electrode containing 0.08 mg MnO_2_ presented specific capacitances of 570 F g^−1^ at 2 A g^−1^ and 271 F g^−1^ at 100 A g^−1^ and maintained 47.5% of its capacitance when increasing the current for 50 times.

Ni–Mn oxyhydroxide was grown on dealloyed nanoporous Ni–Mn alloy by electrochemical oxidation of a Ni–Mn alloy in alkaline media.^86^ The oxyhydroxide developed into the nanopore channels, via the paradigm of domain matching epitaxy, with a small lattice mismatch by dislocations. This electrode possessed efficient electron transport to the hydroxide layer and delivered volumetric capacitance of 505 F cm^−3^ at 0.5 A cm^−3^ and response rate with capacitance of 339 F cm^−3^ at 10 A cm^−3^. Ni–Co oxyhydroxide nanoplates were formed over vertically grown Ni–Co 3D dendrites by electrooxidation processes in alkaline media.^87^ This architecture provided a large number of active sites for redox reactions, enhanced the contact of active materials with conducting channel, favored the diffusion of electrolyte ions, and possibly accommodated volume expansion/contraction phenomena. As a result, the electrode delivered capacity of 121 mAh g^−1^ at 5 mV s^−1^ with acceptable cycle life. Electrodeposition of Co(OH)_2_ on nanoporous Au^88^ resulted in electrodes that achieved specific capacity of 1800 F g^−1^ at 20 A g^−1^. Ni(OH)_2_–nanoporous Au was fabricated by chemically dealloying of electrodeposited Ag_30_Au_70_ followed by hydrothermal growth of Ni(OH)_2_, forming corral‐like Ni(OH)_2_ films on the top of the nanoporous Au and thin Ni(OH)_2_ layer in the inner pores (Figure 10k,l).^89^ The maximal capacity was obtained by optimal growth of Ni(OH)_2_ on the top and in the inner pores of nanoporous Au, which lowered the interface resistance to result in high gravimetric and volumetric capacities of 3168 F g^−1^ and 2223 F cm^−3^, respectively, at 5 A g^−1^ (Figure 10m). Moreover, this electrode also achieved very high response rate, with 70% capacity retention at 500 A g^−1^ (Figure 10m), and long‐term stability with capacity loss of 10% after 30 000 charge–discharge cycles. These interesting results were obtained not only by the formation of the nanoporous Ni(OH)_2_–Au, but also by lowering the interfacial resistance between Ni(OH)_2_ (p‐type semiconductor) and Au due to low Ohmic contact as a result of their matching work function. In fact, the work function of Ni(OH)_2_ is 4.97 eV and that of Au is 5.1 eV. Thus, Ni(OH)_2_–Au formed a matched contact (Figure 10l).

**Figure 11:**
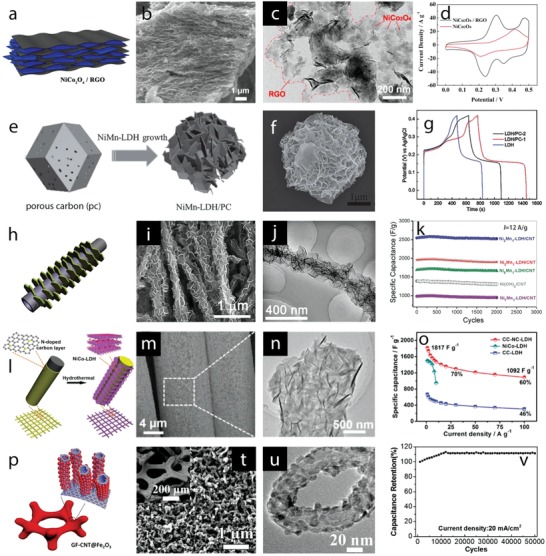
Schematic illustrations, FEG‐SEM and TEM images, and electrochemical performance of different metal oxide/hydroxide–carbon composites/hybrids. a–d) NiCo_2_O_4_–graphene hybrid; e–g) Ni–Mn hydroxide–porous carbon composite; h–k) Ni–Mn hydroxide–carbon nanotube hybrid; l–o) Ni–Co hydroxide–N‐doped carbon–carbon cloth composite; p–v) Fe_2_O_3_–carbon nanotube–graphite foam composite. d) Cyclic voltammograms at 20 mV s^−1^ of NiCo_2_O_4_ and NiCo_2_O_4_–graphene hybrid; g) charge–discharge curves at 1 A g^−1^ of Ni–Mn hydroxide and its composite with porous carbon; k) capacity values of Ni(OH)_2_ and Ni–Mn hydroxides on carbon nanotube composites; o) capacity values at different specific currents of Ni–Co hydroxide, Ni–Co hydroxide–carbon cloth, and Ni–Co hydroxide–N‐doped carbon–carbon cloth composite; v) capacity retention of Fe_2_O_3_–carbon nanotube–graphite foam composite during 50 000 charge–discharge cycles. a–d) Reproduced with permission.^92^ Copyright 2017, Elsevier B.V. e–g) Reproduced with permission.^97^ Copyright 2017, John Wiley and Sons. h–k) Reproduced with permission.^99^ Copyright 2014, John Wiley and Sons. l–o) Reproduced with permission.^100^ Copyright 2017, John Wiley and Sons. p,t,u,v) Reproduced with permission.^101^ Copyright 2015, American Chemical Society.

Graphene is currently among the most studied carbon materials for composite fabrication and hybridizing with metal compounds, particularly oxides and hydroxides. On the one hand, graphene confers interesting physicochemical properties that are suitable to enhance electroactivity of metal oxides and hydroxides, to increase specific surface area, to enhance electrical conductivity, and to provide high flexibility and high mechanical strength. On the other hand, graphene is the most recent discovered carbon form and it has been intensively investigated, a fact that resulted in important advancements concerning new applications. Composites or hybrid materials made of metal oxides or hydroxides with graphene in different forms have been reported for aqueous supercapacitor electrodes. Fabrication routes include dispersion or wrapping on/with graphene, exfoliation and restacking to form multilayer hybrid materials, or coating on (or with) 3D graphene films. Fe_2_O_3_–graphene hydrogel composites were prepared by a hydrothermal method, where Fe_2_O_3_ simultaneously grew together with graphene hydrogel, leading to uniform dispersion of Fe_2_O_3_ on the graphene sheets.^90^ This hydrogel composite showed higher specific surface area (173 m^2^ g^−1^) compared to individual graphene hydrogels (134 m^2^ g^−1^) and Fe_2_O_3_ particles (24 m^2^ g^−1^), thus increasing the number of sites available for the redox reactions and favoring electrolyte diffusion. Furthermore, the conducting graphene network enhanced electron transport into Fe_2_O_3_ and prevented dissolution and agglomeration of Fe_2_O_3_ nanoparticles. The Fe_2_O_3_–graphene hydrogel composite electrode displayed higher capacity and response rate compared to graphene hydrogel and Fe_2_O_3_ nanoparticles, delivering a specific capacity of 908 F g^−1^ at 2 A g^−1^ and good response rate with 69% capacity retention at 50 A g^−1^. The charge–discharge stability of the composite was enhanced compared to Fe_2_O_3_ nanoparticles. RuO_2_–graphene hybrid was prepared by disassembly/reassembly of graphene monoliths to load RuO_2_ particles inside the monolith, forming a uniform high‐density material. This high‐density electrode delivered a high volumetric capacitance (1485 F cm^−3^ at 0.1 A g^−1^).^91^ Layered NiCo_2_O_4_–reduced graphene oxide (rGO) hybrids were prepared by exfoliation and layer‐by‐layer assembly of Ni–Co hydroxide and graphene oxide (GO) followed by freeze drying and thermal treatment.^92^ This architecture (**Figure** **11**a–c) contains a 3D conductive graphene network that could suppress restacking of graphene and reaggregation of NiCo_2_O_4_, resulting in increased specific surface area (167.6 m^2^ g^−1^ vs 109.7 m^2^ g^−1^ of NiCo_2_O_4_). The redox activity was enhanced (Figure 11d), and the electrode delivered specific capacity of 1388 F g^−1^ at 0.5 A g^−1^, good response rate (840 F g^−1^ at 30 A g^−1^), and long‐term charge–discharge stability with 90.2% capacity retention after 20 000 cycles.

β‐Ni(OH)_2_/graphene hybrids were prepared by simple solvothermal reaction of a Ni precursor, GO, and water in benzyl alcohol.^93^ By controlling the amount of Ni precursor, in low quantity, single layer‐by‐layer assembly of β‐Ni(OH)_2_ and graphene was formed. Amorphous FeOOH quantum dots dispersed over functionalized graphene sheets were prepared by hydrothermal reaction and displayed good charge storage performance.^94^ Ni(OH)_2_–graphene hybrid hydrogel was prepared by a hydrothermal method.^95^ The graphene hydrogel was constructed of out‐of‐plane pores and contained in‐plane pores, which formed a hierarchical pore structure, providing efficient sites to anchor Ni(OH)_2_ and favoring access of electrolyte. The charge storage performance of Ni(OH)_2_–graphene hybrid outperformed its single components and the electrode achieved capacity of 1250.3 F g^−1^ at 5 A g^−1^. Ni–Co hydroxide–graphene monolithic composite electrodes were fabricated by a hydrothermal method, followed by vacuum filtration and contained alternate layers of NiCo–CH nanowires and graphene nanosheets.^96^ Oxygen species on graphene sheets supported the nucleation and growth of well‐dispersed Ni–Co hydroxide nanowires, which formed open porous channels between the graphene sheets, leading to high charge storage performance. Carbon nanotubes were also introduced in the composite, acting as conducting linkers between graphene sheets and Ni–Co hydroxide. Carbon nanotubes increased the mass and volume of the electrode, decreasing the specific capacity, but the enhanced electrical conductivity (16.8 S m^−1^ for the electrode with carbon nanotubes vs 4.2 S m^−1^ for the electrode without carbon nanotubes) improved the response rate.

Other forms of carbon nanostructures have also been reported for composites with metal oxides and hydroxides. Ni–Mn hydroxide–porous carbon composites were prepared by the hydrothermal method.^97^ Porous carbon was carbonized from zeolitic imidazolate framework‐8 (ZIF‐8), forming a regular rhombic dodecahedral carbon shape. The deposition of Ni–Mn hydroxide uniformly covered the porous carbon, which surrounded the carbon with interlinked and disordered nanosheets (Figure 11e,f). This composite displayed mesoporous structure and enhanced electrical conductivity, and the agglomeration of Ni–Mn hydroxide was prevented by the presence of porous carbon, leading to an optimal capacity of 686.28 C g^−1^ at 1 A g^−1^ (Figure 11g). NiCo_2_O_4_–porous carbon composites were also prepared using ZIF‐8‐derived porous carbon and Ni–Co precursors followed by thermal transformation, and the electrode showed good charge storage performance.^98^ Ni–Mn hydroxide–carbon nanotube shell–core hybrids were prepared by a coprecipitation technique, forming gauze‐like NiMn‐LDH nanosheets attached along the nanotube backbone (Figure 11h–j).^99^ The hybrids with carbon nanotubes provided an endurance framework for grafting the hydroxide and their contact boosted electron transfer to the hydroxide layer. Moreover, this hybrid possessed a high specific surface area (198 m^2^ g^−1^) and pore volume (0.38 cm^3^ g^−1^), resulting in high‐performance electrodes (Figure 11k). Ni–Co hydroxide–N‐doped carbon–carbon cloth was prepared through dip coating and carbonization to form N‐doped carbon on the carbon cloth, followed by the hydrothermal growth of an interconnected Ni–Co hydroxide nanosheet layer (Figure 11l–n).^100^ The N‐doped carbon layer improved the hydrophilicity of the carbon cloth (contact angle decreased from 135° to 0° after coating with N‐doped carbon), favored the nucleation and growth of Ni–Co hydroxide layer, and formed a strong electronic interaction with the hydroxide layer. The composite electrode retained the superhydrophilic property, with contact angle of 0°, thus favoring the electrolyte diffusion and enhancing the electrode electrochemical response. These properties contributed to build electrode with high capacity (1817 F g^−1^ at 1 A g^−1^, above that of Ni–Co hydroxide on the carbon cloth, Figure 11o), high response rate, and long cycle life. A Fe_2_O_3_–carbon nanotube–graphite foam composite, composed of Fe_2_O_3_ nanoparticles grafted on a 3D graphite foam–carbon nanotube nanoforest collector, was prepared via two‐step deposition.^101^ The carbon nanotube nanoforest was grown on the graphite foam by chemical vapor deposition, to form a composite foam collector, and Fe_2_O_3_ nanoparticles were grown on this modified carbon foam by atomic layer deposition (Figure 11p,t,u,v). The composite carbon foam collectors possessed high surface area, high conductivity, and good flexibility, and provided not only the double‐layer charge storage, but also anchored sites for the growth of Fe_2_O_3_ and electrical contacts to increase electron transfer in the intrinsically poor conductive Fe_2_O_3_. This composite electrode achieved high areal capacity (470.5 mF cm^−2^ at 20 mA cm^−2^) and high charge–discharge stability up to 50 000 cycles (Figure 11v).

Composites/hybrids of different metal oxides and hydroxides have also been studied, to take advantages of different redox reactions in distinct active materials in the composites or to design hierarchical structures to enhance electroactive surface and electrolyte diffusion. Such composites of MnO_2_–NiCo_2_O_4_,^102^ MnCo_2_O_4_–Ni(OH)_2_,^103^ Mn–Co hydroxide–Ni(OH)_2_,^104^ Fe–Co hydroxide–NiO,^105^ and TiO_2_–Ni(OH)_2_ have shown good charge storage performance and promising properties as electrode materials for supercapacitors.^106^

## 4 Tailored Devices

Materials engineering and different functionalization approaches have been proposed to fabricate electrodes for supercapacitors with increased charge storage capacity (much above the values typical of the double layers on carbon electrodes), high power response, and long‐term operation. Typically, in carbon‐based double‐layer supercapacitors, cells are assembled in the symmetric configuration, which consist of two similar capacitive electrodes (negative electrode and positive electrode) isolated by a separator immersed in the electrolyte. This assembly limits the working voltage of the devices to the working potential of the symmetric cell. The energy density of the device is proportional to the integrated area under the charge–discharge curve, which pinpoints the importance of enlarging the working voltage. Thus, asymmetric designs are attracting a lot of attention. These devices consist of two different charge storage electrodes, electrochemically active in different potential windows, that when combined result in wider working voltage. Moreover, the complementary water decomposition potential of each electrode, with different overpotentials for hydrogen and oxygen evolution, can widen the working voltage of the cell, over the theoretical water splitting potential range of 1.23 V. Therefore, asymmetric assemblies are envisioned as an important route to boost energy density of aqueous supercapacitors.

It is worth noting that supercapacitors are power devices. The state of the art in developing high energy density electrodes is not only by fabricating capacitive and pseudocapacitive charge storage materials, but also by producing battery‐like and hybrid (mixed battery and capacitive responsive) responsive materials to maximize power. Thus, in the asymmetric design, to keep the maximum power response, capacitive carbon materials are used in one of the electrodes, despite the fact that energy density is modest due to limited double‐layer charging. A well‐known example is the Li supercapacitor, a device that still requires lithiation steps and organic electrodes.

Metal oxides and hydroxides can store charge in either more positive or more negative regions of the potential window. Thus, it is possible to combine different metal oxides and hydroxides, with enhanced redox kinetics, in positive and negative electrodes to deliver high charge storage capacity, good power response, and long‐term stability. These asymmetric combinations can improve much further energy density of supercapacitor cells, while maintaining adequate power response, even in aqueous electrolytes. It is worth noting that asymmetric designs employing battery‐like responsive electrodes shall be referred to as hybrid supercapacitors.

**Figure 12:**
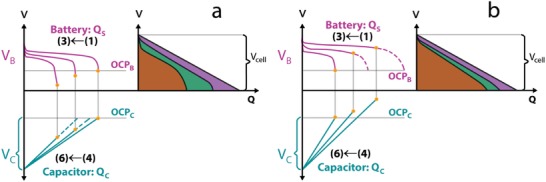
Discharge curves of the battery‐type electrodes (1–3), the capacitive electrodes (4–6), and the corresponding asymmetric cells at three currents with charge balancing at a) low and b) high currents. a,b) Reproduced with permission.^107^ Copyright 2015, Elsevier B.V.

Charge balancing between the positive electrode and the negative electrode is required when assembling asymmetric supercapacitor cells. **Figure** **12** shows the discharge profile of asymmetric cells assembled with electrodes that make use of materials with different charge storage behavior (capacitive and battery‐like). The capacity of the asymmetric cell at high currents depends on whether the charge is balanced at low or high currents due to the different response rates of negative and positive electrodes (Figure 12).^107^ Although charge balancing is widely applied, recently it has been shown that charge unbalancing, interestingly, could widen the working voltage of the symmetric cell, an issue that certainly will be further investigated.^108^

Traditionally, aqueous electrolytes, including acidic (e.g., H_2_SO_4_, HNO_3_), neutral (e.g., Na_2_SO_4_, Li_2_SO_4_, KCl), and alkaline (e.g., KOH, NaOH, LiOH), in the liquid form are used in supercapacitor cells. Operation in aqueous electrolytes is of utmost relevance, but there are still some drawbacks to overcome, such as leakage and material corrosion. To avoid that, quasi‐solid‐state gel electrolytes have emerged recently as an interesting option. In these systems, the aqueous electrolyte is trapped in polymer matrices such as poly(vinyl alcohol) (PVA), poly(acrylic acid), and poly(methyl methacrylate), supplying ions for redox reactions with active materials. Quasi‐solid‐state gel electrolytes can prevent dissolution or corrosion of active materials and the leakage of electrolyte, improving lifetime of devices. Moreover, assembling cells using quasi‐solid‐state gels offers advantages of flexibility, safety, and wider working temperate range,^50^ advancing cell package engineering for functional solid‐state supercapacitors.

Supercapacitors displaying high energy density are presently a very important target and, nowadays, many applications have specific requirements, demanding specially tailored supercapacitors (Figure 1, device assembly axis). For example, development of miniaturized devices and flexible and wearable electronics imposes integration of reliable micro‐supercapacitors and/or flexible supercapacitors.

Integration of supercapacitors with renewable electrical energy sources, such as solar cells and wind turbines, and replacement of conventional Al electrolytic capacitors require devices with short time response, leading to the development of high‐frequency micro‐supercapacitors. The replacement of multicomponents with multifunctional devices can save space, which is of utmost relevance for electronics, leading to the development of supercapacitors tailored for multiple purposes. During operation of supercapacitors, irreversible deformations or failures could occur due to intentional bending, twisting, and stretching or accidental causes. Thus, supercapacitors with restorable abilities, such as healable or shape memory to heal failures or to recover back to the original shape, are also being developed.

The following subsections address the latest advances in metal oxide and hydroxide–based aqueous supercapacitors tailored to answer the application needs. For simpler description, supercapacitor cells are denoted as negative electrode material||positive electrode material. These subsections are organized as follows. First, typical carbon||metal oxide/hydroxide supercapacitors are described followed by new metal oxide/hydroxide||metal oxide/hydroxide supercapacitors with enhanced energy density. Then, micro‐supercapacitors including high‐frequency ones and flexible supercapacitors will be discussed. Finally, some advances on multipurpose supercapacitors, and restorable and degradable supercapacitors will be discussed.

### 4.1 High Energy Density Supercapacitors

#### 4.1.1 Carbon||Metal Oxide/Hydroxide Supercapacitors

Many metal oxides and hydroxides, such as NiCo_2_O_4_, MnO_2_, Ni(OH)_2_, and Co(OH)_2_, display redox response in aqueous electrolytes in positive potential windows. Different materials engineering routes, as described previously, allow maximizing the redox response, making metal oxides and hydroxides suitable materials to assemble high energy density electrodes for aqueous supercapacitors. They can be assembled with capacitive carbon electrodes and various carbon‐based materials (e.g., activated carbon, graphene, carbon nanotubes, or their composites), which provide high conductivity and high specific surface area for double‐layer charging. Generally, such configurations display wider working voltages, about 1.6–1.8 V; values near 2 V were also achieved.

**Figure 13:**
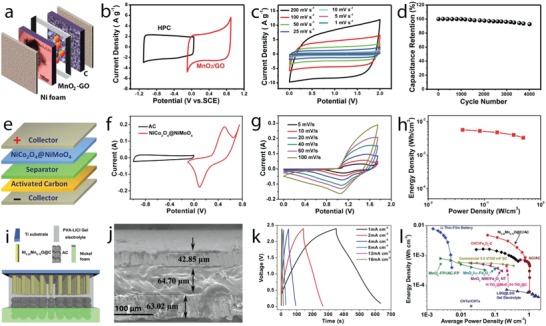
High energy density carbon||metal oxide aqueous supercapacitors: a–d) porous carbon||MnO_2_–graphene oxide; e–h) activated carbon||NiMoO_4_–NiCo_2_O_4_ and i–l) activated carbon||Ni_0.25_Mn_0.75_O @ C. a,e,i) Schematic illustrations of the supercapacitors; b,f) cyclic voltammograms of carbon negative electrodes and metal oxide positive electrodes; c,g) cyclic voltammograms at different scan rates; d) capacitance retention of porous carbon||MnO_2_–graphene oxide supercapacitor during 4000 charge–discharge cycles; h) Ragone plot of activated carbon||NiMoO_4_–NiCo_2_O_4_ supercapacitors; j) cross‐sectional FEG‐SEM image and k) charge–discharge plots at different current densities and Ragone plot of the solid‐state activated carbon||Ni_0.25_Mn_0.75_O @ C supercapacitors (Ragone plot included other supercapacitors for comparison). a–d) Reproduced with permission.^111^ Copyright 2014, John Wiley and Sons. e–h) Reproduced with permission.^116^ Copyright 2015, Royal Society of Chemistry. i–l) Reproduced with permission.^118^ Copyright 2017, John Wiley and Sons.

Asymmetric cells employing pseudocapacitive materials, such as manganese oxide, can display increased energy density and high power density as a result of the pseudocapacitive response of the oxide and capacitive response of carbon, respectively. Several asymmetric cells based on this configuration and delivering high capacitive performance have been reported: MnO_2_ nanotube–activated graphene,^109^ MnO_2_–carbon nanofiber composites,^110^ and MnO_2_–graphene oxide composites.^111^ Porous MnO_2_ nanotubes consisting of MnO_2_ nanosheets were prepared by hydrothermal routes, using polycarbonate membranes as sacrificial template.^109^ When assembled into cells using activated graphene as negative electrodes, in 1 m Na_2_SO_4_ aqueous electrolytes, activated graphene||MnO_2_ nanotubes delivered energy density of 22.5 Wh kg^−1^ and maximum power density of 146.2 kW kg^−1^ in working voltage windows of 1.8 V. MnO_2_–carbon nanofiber composites were prepared by carbonization of bacterial cellulose pellicles, followed by redox reactions of MnO_4_^−^ with carbon to form coatings made of MnO_2_ nanoparticles on the surface of carbon nanofibers.^110^ MnO_2_–carbon nanofiber as positive electrode was assembled with N‐doped carbon nanofiber (prepared by carbonization of bacterial cellulose pellicles in the presence of urea) as negative electrodes and the devices could operate in working voltage windows of 2 V. This N‐doped carbon nanofiber||MnO_2_–carbon nanofiber cells delivered energy density of 32.91 Wh kg^−1^ and maximum power density of 284.63 kW kg^−1^, as well as good cycle life, with 95.4% capacity retention after 2000 cycles. Hierarchical porous carbon||MnO_2_–graphene oxide cells were prepared combining nanoflaked MnO_2_–graphene oxide multilayers and hierarchical porous carbon prepared by carbonization of natural *Artemia* cyst shells (**Figure** **13**a).^111^ These cells operated in working voltage windows of 2 V (Figure 13b,c), delivering energy densities of 46.7 and 18.9 Wh kg^−1^ at power densities of 100 and 2000 W kg^−1^, respectively, and evidenced good cycle life with 93% capacity retention after 4000 cycles (Figure 13d).

Asymmetric cells assembled with battery‐like responsive materials with high storage capacity in the positive electrode are currently an important route to obtain high energy density aqueous supercapacitors. For example, N‐doped graphene||NiO,^50^ reduced graphene oxide||Co*_x_*Ni_1−_ *_x_*O–reduced graphene oxide,^112^ activated carbon nanorod||NiCo_2_O_4_,^113^ activated carbon||NiMoO_4_,^114^ activated carbon||Mg‐doped α‐Ni(OH)_2_,^76^ activated carbon||Ni–Co hydroxide–N‐doped carbon,^100^ and carbon nanofoam paper (CNFP)||Ni*_x_*Co_1−_ *_x_*(OH)_2_–CNFP cells have been proposed.^115^ It is interesting to note that despite battery‐like responsive materials, with well‐defined redox peaks, being employed as positive electrodes, the resulting asymmetric cells could display quasi‐rectangular cyclic voltammograms, suggesting good capacitive behavior.^112^, ^114^ Those supercapacitor cells generally work in alkaline electrolytes since the currently studied battery‐like responsive electrodes typically display optimal redox response in such media. The low overpotential for oxygen evolution of those positive electrodes and the low overpotential for hydrogen evolution of carbon materials in alkaline media limit the working voltage of cells to about 1.5–1.7 V. Activated carbon||NiMoO_4_–NiCo_2_O_4_ cells (using NiMoO_4_ nanosheets shell–NiCo_2_O_4_ nanowire core arrays as positive electrode) operated in a voltage window of 1.75 V in 2 m KOH (Figure 13e–g) and delivered volumetric energy density of 5.64 mWh cm^−3^ at power density of 0.04 W cm^−3^ (Figure 13h).^116^ Activated carbon||Mg‐doped α‐Ni(OH)_2_ cells operated in working voltage windows of 1.6 V in 6 m KOH and delivered maximum energy and power densities of 57.9 Wh kg^−1^ and 26 kW kg^−1^, respectively.^76^ Activated carbon||Ni–Co hydroxide–N‐doped carbon cell operated in voltage windows of 1.6 V and displayed energy densities of 69.7 and 41.5 Wh kg^−1^ at power densities of 0.8 and 21.6 kW kg^−1^, respectively, and long life cycle up to 20 000 cycles.^100^

Quasi‐solid‐state asymmetric cells based on carbon||metal oxide/hydroxide are also commonly reported, being based on activated carbon||CoMoO_4_/CoNiO_2_ and carbon nanotube||Co_3_O_4_–CoMoO_4_ cells working in LiOH/KOH–PVA gel electrolyte.^117^ Activated carbon||CoMoO_4_/CoNiO_2_ cells using CoMoO_4_/CoNiO_2_ core/shell nanowires/nanoneedles grown on Ni foam by a two‐step hydrothermal route were used as positive electrode.[[qv: 117a]] This device displayed energy densities of 59.75 and 11.33 Wh kg^−1^ at power densities of 1464 and 14 880 W kg^−1^, respectively, in a voltage window of 1 V, with no significant capacity loss after 50 000 cycles. A carbon nanotube||Co_3_O_4_–CoMoO_4_ cell was assembled with a positive electrode made of CoMoO_4_ nanosheets grown on Co_3_O_4_ nanocones/Ni foam via a two‐step hydrothermal route.[[qv: 117b]] It delivered energy densities of 45.2 and 37.0 Wh kg^−1^ at power densities of 400 and 6400 W kg^−1^, respectively, within the working voltage window of 1.6 V and the capacity retention was 96.5% after 3000 cycles. These quasi‐solid‐state asymmetric cells displayed capacitive response, evidencing quasi‐rectangular cyclic voltammograms and promising application as hybrid supercapacitors. Quasi‐solid‐state asymmetric cells working under voltage windows up to 2.4 V have also been described.^118^ This design was based on the formation of Ni_0.25_Mn_0.75_O @ C electrode with high oxygen evolution potential and displayed electrochemical response in the potential range of 0–1.4 V. Thus, an asymmetric cell made of activated carbon||Ni_0.25_Mn_0.75_O @C in LiCl–PVA electrolyte (Figure 13i,j) could operate in a working voltage window of 2.4 V (Figure 13k), delivering high energy and power densities (Figure 13l).

#### 4.1.2 Metal Oxide/Hydroxide||Metal Oxide/Hydroxide Supercapacitors

The energy density of asymmetric supercapacitor cells is limited by the lowest capacity electrode. In the case of carbon||metal oxide/hydroxide cells, carbon electrodes with lower capacity reduce the energy density of the whole cell. To overcome this limitation, asymmetric cells that employ redox materials in both electrodes have been proposed for high energy density supercapacitors. Thus, it is essential to develop active materials, for both the positive and negative electrodes with well‐matched redox potentials to enable optimal energy performance. This is actually a very dynamic research line and paves the way to design new supercapacitor assemblies matching increased energy and power densities.

**Figure 14:**
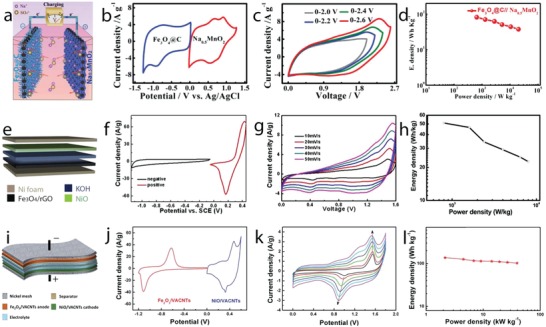
High energy density metal oxide||metal oxide supercapacitors: a–d) Fe_3_O_4_@C||Na_0.5_MnO_2_, e–h) Fe_3_O_4_/rGO||NiO, i–l) Fe_2_O_3_/vertically aligned carbon nanotubes (VACNTs)||NiO/VACNTs. a,e,i) Schematic illustrations of the supercapacitors, b,f,j) cyclic voltammograms of the negative electrodes and the positive electrodes, c,g,k) cyclic voltammograms at different working voltages or different scan rates, and d,h,l) Ragone plot of the supercapacitors. a–d) Reproduced with permission.^121^ Copyright 2017, John Wiley and Sons. f–h) Reproduced with permission.[[qv: 122a]] Copyright 2016, Springer Nature. i–l) Reproduced with permission.^125^ Copyright 2016, Royal Society of Chemistry.

Pseudocapacitive materials store charge via fast and reversible redox reactions, mimicking the capacitive response of carbon‐based materials. When using pseudocapacitive materials for both electrodes in asymmetric cells, their fast kinetic redox reactions can provide simultaneously high power response and high energy density. For example, FeWO_4_||MnO_2_ (both electrodes are pseudocapacitive) were assembled in an asymmetric coin cell, working in 5 m LiNO_3_ electrolyte.^119^ This cell stored charge in a voltage range of 1.4 V and displayed specific capacitance of 8 F g^−1^, stable charge and discharge over 35 000 cycles, good Coulombic efficiency (nearly 100% over 35 000 cycles), low leakage current, and low self‐discharge rate. α‐Fe_2_O_3_ nanoneedle/Ni nanotube||MnO_2_ nanosheet/Ni nanotube cells operated in Na_2_SO_4_ aqueous electrolyte or Na_2_SO_4_/PVA poly‐mer gel electrolyte^82^ and delivered better redox performance due to the higher ionic conductivity of the liquid form. These cells reached maximum energy density of 34.1 Wh kg^−1^ at power density of 3197.7 W kg^−1^, and were stable up to 5000 cycles, without significant capacity loss. Fe_3_O_4_@Fe_2_O_3_||Fe_3_O_4_@MnO_2_ cells were assembled using core–shell structures consisting of Fe_2_O_3_ or MnO_2_ grown over Fe_3_O_4_ nanorod cores.^120^ These cell were operated in a working voltage window of 2 V and achieved energy densities of 83 mWh cm^−3^ (26.6 Wh kg^−1^) and 0.45 mWh cm^−3^ (14.5 Wh kg^−3^) at power densities of 15.6 mW cm^−3^ (500 W kg^−1^) and 500 mW cm^−3^ (16 kW kg^−1^), respectively. Using this design concept, supercapacitor cells with working voltage windows up to 2.6 V were able to reach the working voltage of organic supercapacitors, as recently reported.^121^ Based on the formation of composite materials, containing carbon cloth with increased overpotential for oxygen and hydrogen evolution in neutral electrolytes, the Na_0.5_MnO_2_/carbon cloth could operate in a working potential range of 0–1.3 V and carbon‐coated Fe_3_O_4_/carbon cloth could operate in a working potential range of −1.3 to 0 V (**Figure** **14**b). Thus, the asymmetric C‐coated Fe_3_O_4_||Na_0.5_MnO_2_ (Figure 14a) cells could store charge in a working voltage window of 2.6 V (Figure 14c),^121^ delivering energy density of 81 Wh kg^−1^ at power density of 647 W kg^−1^ and retaining 93% of the initial capacity over 10 000 cycles (Figure 14d).

Asymmetric cells made of pseudocapacitive materials combined with battery‐like responsive materials are also being studied, following the concept of carbon||battery‐like responsive material cells. For example, Fe_3_O_4_/reduced graphene oxide||NiO and FeOOH||NiMoO_4_ cells have been reported, showing high charge storage performance.^122^ Fe_3_O_4_/reduced graphene oxide||NiO cells were assembled using triple‐shelled NiO hollow microspheres as battery‐like responsive electrode and Fe_3_O_4_ nanoparticles dispersed over reduced graphene oxide sheets as pseudocapacitive electrode in KOH electrolyte (Figure 14e). This cell stored charge over a working voltage of 1.6 V (Figure 14f,g) and delivered energy density of 51.0 Wh kg^−1^ at a power density of 800 W kg^−1^ (Figure 14h). FeOOH||NiMoO_4_ cells were assembled using low‐crystalline FeOOH nanoparticles as pseudocapacitive electrode and NiMoO_4_ nanowires as battery‐like electrode. The FeOOH nanoparticles presented high specific capacitance of 1066 F g^−1^ at 1 A g^−1^ at mass loadings of 1.6 mg cm^−2^; even at high mass loading of 9.1 mg cm^−2^, which is comparable with the mass loading of commercialized carbon supercapacitor, they still delivered specific capacitance of 716 F g^−1^ (186 F cm^−3^) at 1 A g^−1^. Thus, these cells could deliver energy densities of 104.3 and 31 Wh kg^−1^ at power densities of 1.27 and 0.94 kW kg^−1^, respectively, in a working voltage window of 1.7 V. A device was assembled and delivered energy densities of 31.44 Wh kg^−1^ (17.24 Wh L^−1^) and 12.72 W kg^−1^ at power densities of 305 and 4.976 W kg^−1^ (2.736 W L^−1^), respectively. This cell design was stable under both charge–discharge cycling and floating tests.

Interestingly, supercapacitor cells assembled with battery‐type electrodes, for both electrodes, have also been reported and displayed good performance. Probably, fast Faradaic redox reactions at each electrode, or the formation of composites with carbon‐based materials, enabled these well‐matched electrochemical responses.^123^ For example, a ZnFe_2_O_4_/stainless steel mesh||Ni(OH)_2_/stainless steel mesh cell in which both electrode have well‐defined redox peaks was recently assembled^124^ in a working voltage window of 1.6 V. The cell delivered energy density of 42 Wh kg^−1^ at power density of 5 kW kg^−1^ and retained 83% of initial capacity after 8000 cycles. Fe_2_O_3_/carbon nanotube||NiO–carbon nanotube cells in a 1.8 V window delivered energy densities of 137.3 and 102.2 Wh kg^−1^ at power densities of 2.1 and 39.3 kW kg^−1^, respectively (Figure 14i–l).^125^ Fe_2_O_3_/carbon nanotube/graphite foam||CoMoO_4_/graphite foam cells were assembled in KOH electrolyte^101^ and worked under the voltage of 1.6 V, retaining 94.5% of their initial capacitance after 50 000 charge–discharge cycles. These cells delivered energy densities of 74.7 and 41.1 Wh kg^−1^ at power densities 1.4 and 11.2 kW kg^−1^, respectively, and displayed good capacitive response, evidencing quasi‐rectangular cyclic voltammograms, due to the fact that both electrodes are carbon‐based composites, favoring the redox reactions at each one.

### 4.2 Micro‐Supercapacitors

Smart electronic devices based on autonomous microsystems have evolved rapidly and presently are used in a wide range of applications. Microsystems and devices such as micro/nanoelectromechanical systems, micro/nanorobots, health sensors (including implant biosensors), and environmental and industrial sensors, with wireless sensing and communication abilities, enabled sensing and transfer of information across networks and advanced the development of smart integration systems. Such miniaturized autonomous devices require electrical energy supply to serve their purpose. Following the use of batteries for large‐scale devices, miniaturized batteries (microbatteries) with a size compatible with microsystems can be integrated in these systems. However, limited power density and lifetime have restricted their use in microsystems, when peak on/off power supply is required (e.g., sensing devices) or when long‐term use is expected (e.g., implant devices). Thus, miniaturized supercapacitors (also known as micro‐supercapacitors), which display energy storage/release characteristics similar to those of macro‐supercapacitors, have been proposed as power supply devices for microsystems. Nevertheless, the development of micro‐supercapacitors is quite behind miniaturized electronic system technologies, opening an interesting research frontier.

**Figure 15:**
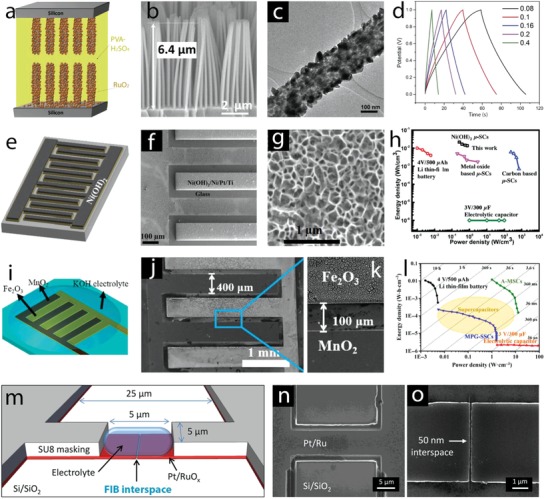
Metal oxide and hydroxide micro‐supercapacitor cells assembled in different configurations: a–d) sandwich symmetric RuO_2_/Si nanowires||RuO_2_/Si nanowires cell, e–h) planar symmetric Ni(OH)_2_||Ni(OH)_2_ cell, i–l) planar asymmetric Fe_2_O_3_||MnO_2_ cell, and m–o) planar symmetric RuO*_x_*||RuO*_x_* cell designed by combining photolithography and focus ion beam techniques. a,e,i,m) Schematic illustrations of the micro‐supercapacitors; b,c) FEG‐SEM and TEM images of Si nanowire arrays and RuO_2_‐coated Si nanowire; f,g,j,k,n,o) FEG‐SEM or TEM images of the micro‐supercapacitors at different magnifications; d) charge–discharge plots of RuO_2_/Si nanowires||RuO_2_/Si nanowire cell; and h,l) Ragone plots of Ni(OH)_2_||Ni(OH)_2_ and Fe_2_O_3_||MnO_2_ cells and their performance comparison with other devices. a–d) Reproduced with permission.^126^ Copyright 2016, Elsevier B.V. e–h) Reproduced with permission.^127^ Copyright 2014, John Wiley and Sons. i–l) Reproduced with permission.^128^ Copyright 2017, Springer Nature. m–o) Reproduced with permission.^129^ Copyright 2017, American Chemical Society.

Micro‐supercapacitor assemblies follow those of conventional electrochemical energy storage cells, including two electrodes and the electrolyte. They are assembled in different designs, frequently as sandwich structures (**Figure** **15**a), in‐plane structures (Figure 15e), and fiber‐shaped structures. The fiber‐shaped structure, which advances the integration of micro‐supercapacitor in flexible microelectronics, is out of the scope of this section and will be discussed in the following section regarding flexible supercapacitors. Sandwich‐like micro‐supercapacitors mimic the design of bulky supercapacitors or thin‐film microbatteries, in which the electrolyte (generally in solid state) is inserted between two parallel charge storage electrodes. This simple design facilitates large‐scale fabrication, but the integration into microdevices is still very challenging. In‐plane design, micro‐supercapacitors are constructed of microelectrodes integrated on a common substrate, which are physically separated by interspacing between them. This design involves the use of microfabrication methods, taking advantage of the current state of the art in microfabrication technologies, to precisely define the architecture pattern of the supercapacitor electrode. Moreover, it makes easier integration of micro‐supercapacitors into microsystems. Note that, in micro‐supercapacitors, both mass and volume per unit area of active material are very small and occupy a nonsignificant part of the whole microsystem. The integration area of the devices in microsystems is the most significant parameter among density and geometric considerations. As a consequence, gravimetric capacity, which is widely reported, becomes an unsuitable metric and the energy storage capacity of the integrated devices per footprint area (areal capacity) is the most important capacity metric for micro‐supercapacitors. The sandwich‐like micro‐supercapacitor, in which each electrode occupies a footprint area, can deliver high areal capacity, if the electrode design enables better diffusion between the electrolyte and electrolyte/electrode contact. However, this design often displays decreased areal energy density compared to the in‐plane design, when using the same electrode materials, due to increased electrolyte diffusion into both the positive and negative microelectrodes, thanks to the interspacing between them.

The use of redox materials based on metal oxides and hydroxides for micro‐supercapacitors can significantly enhance energy density. Nevertheless, electrode materials engineering is still required to increase surface to volume ratio and is extremely important to enlarge areal capacity. Microfabrication techniques such as printing and lithography followed (or not) by deposition or post‐processing method to form redox‐active metal oxides and hydroxides are being used to fabricate in‐plane micro‐supercapacitors. Nevertheless, microfabrication techniques to precisely control the interspacing down to nanosize and to increase the footprint areas of electrodes still require important development to enhance the electrode areal capacity.

Based on micropatterning of active materials, their precursors, or current collectors (followed by depositing of active materials), the formation of active materials on microelectrodes could favor the fabrication processes. Thus, symmetric designs (which were not discussed in the previous section of this review) have been employed to construct micro‐supercapacitors. The next section highlights some advances in metal oxide and hydroxide–based micro‐supercapacitors.

#### 4.2.1 Metal Oxide and Hydroxide in Micro‐Supercapacitors

To facilitate the integration of micro‐supercapacitors with electronic components on the same chip, the fabrication processes should be compatible with complementary metal–oxide–semiconductor processes that are used for construction of the integrated circuits. Thus, composites of redox materials with engineered porous Si substrate are considered the best choice to enhance charge storage performance. For example, sandwich micro‐supercapacitors were assembled in PVA–H_2_SO_4_ gel electrolyte using an atomic layer deposited coating of RuO_2_ on Si nanowire arrays prepared by metal‐assisted anodic etching of commercial wafers (Figure 15a–c). The device showed areal capacitance 2.9 mF cm^−2^ at 0.08 mA cm^−2^ and 2.3 mF cm^−2^ at 0.4 mA cm^−2^ (Figure 15d) and long‐term stability.^126^ It should be noted that despite the high cost of RuO*_x_* that hinders its wide use in bulky high energy density supercapacitors, the low quantity required in micro‐supercapacitors does not raise the costs significantly, since the main cost is mainly the consequence of the microfabrication processes. Sandwich symmetric RuO_2_·*x*H_2_O–Au foam cells assembled in silicotungstic acid–PVA gel electrolyte were integrated in a Si water.^83^ This micro‐supercapacitor delivered a remarkable areal capacitance of 1220 mF cm^−2^ at 1.5 mA cm^−2^, thanks to the formation of the porous hybrid architecture discussed in the previous section.

Presently, the main approach in micro‐supercapacitors involves the design of planar configurations, with current collectors patterned on the substrate by microfabrication and deposition or post‐processing methods, to form the redox‐active materials, which enables easier integration into microsystems. For example, Ni(OH)_2_‐based planar micro‐supercapacitors have been reported (Figure 15e–g).^127^ The fabrication route involved photolithography processes accompanied with metal current collector coating (layered Ni/Pt/Ti) by sputtering deposition and chemical bath deposition of Ni(OH)_2_ layer to obtain interdigitated microelectrode fingers (Figure 15f). This micro‐supercapacitor when operating in KOH electrolyte delivered energy density of 21 mWh cm^−3^ at power density of 262.5 mW cm^−3^ (Figure 15h).

Planar micro‐supercapacitors based on the asymmetric coupling of two redox‐active materials, as in the case of macro‐supercapacitors, have also been reported to enlarge the working potential window and to increase energy density.^128^ The materials used in the asymmetric cell were MnO_2_ as positive electrode and Fe_2_O_3_ as negative electrode (Figure 15i). The electrodes were fabricated on Si wafer by photolithography followed by physical vapor deposition of Cr/Ni layered current collectors and electrodeposition and thermal annealing to form MnO_2_ and Fe_2_O_3_ layers (Figure 15j,k). This on‐chip micro‐supercapacitor in KOH stored charge in a voltage window of 1.2 V and delivered energy densities of 12 mWh cm^−3^ and 35 µWh cm^−3^ at power densities of 1 and 14.8 W cm^−3^ (Figure 15l).

Photolithography processes are currently employed for microfabrication of planar micro‐supercapacitors. However, the resolution of the pattern (interelectrode spacing) is low due to the light diffraction limit. Presently, photolithography processes are used to fabricate planar micro‐supercapacitors with interspacing size above 10 µm over footprint area of 1 cm^2^. For highly integrated planar micro‐supercapacitors, interelectrode spacing should be as small as possible; this issue is still limiting the use of photolithography. Recently, photolithography was combined with focus ion beam techniques to scribe a high‐resolution pattern for RuO*_x_*‐based micro‐supercapacitors (Figure 15m).^129^ This fabrication procedure involved evaporation deposition of Cr/Pt (40/200 nm) layers on Si/SiO_2_ and subsequent sputtering deposition of Ru film. Via optimal photolithography and ion beam etching processes, a high‐resolution pattern with interelectrode spacing down to 50 nm could be achieved (Figure 15n,o). Finally, RuO*_x_* layer was formed by thermal oxidation of the Ru film. This micro‐supercapacitor could operate in H_2_SO_4_ being stable over 10 000 cycles; it also showed very low equivalent series resistance of 8.2 µΩ cm^−2^ and areal capacitance of 18 mF cm^−2^, thanks to the narrow interspacing between the positive and negative microelectrodes.

#### 4.2.2 High‐Frequency Micro‐Supercapacitors

Presently, supercapacitor studies are focusing on the reduction of the energy density gap between supercapacitors and batteries; yet, there is another gap, which is related to the frequency response between supercapacitors and Al electrolytic capacitors. Al electrolytic capacitors are currently being used for voltage ripple filtering when AC is converted to DC in electronic devices as well as for pulse power filtering produced by environmental energy harvesting. The voltage ripple can reduce or fasten the degradation of electronic devices and other energy storage devices. For example, it has been shown that the degradation of batteries is quicker when the charging sources contain voltage ripple.^130^ The standard AC line frequency is 60 Hz and the pulse powers are typically in the frequency range of tens to hundreds Hz, which requires capacitive response at frequencies of 120 Hz or tens to hundreds Hz, respectively. Commercial Al electrolytic capacitors display capacitive response at frequencies from tens to hundreds kHz with large phase angle up to −85.5°, fulfilling the requirement as voltage ripple filtering in most of the electronic devices and the pulse powers from environmental energy harvesting. However, Al electrolytic capacitors are too bulky, which are among the largest components occupying high volume and area in electronic circuits and, overall, they deliver very low energy density. Thus, smaller size AC lines or high‐frequency power filtering supercapacitors are being developed to integrate with emerging miniaturized electronic devices. Most of supercapacitors can only store charge at frequencies below 1 Hz with resistor–capacitor (RC) time constant of about 1 s, thus lacking voltage ripple filtering abilities to produce pure DC voltage. At high frequencies, most of supercapacitors behave like resistors with phase angles close to 0° due to their high electronic and ionic resistances, which does not comply with the phase angle requirement of −90° for AC line‐filtering supercapacitors. John Miller has first produced AC line‐filtering micro‐supercapacitors by chemical vapor deposition of graphene films, consisting of vertically aligned and interlinked graphene sheets on a metallic current collector.^131^ The symmetric supercapacitors when operating in 1 m KOH aqueous electrolyte showed phase angle at 120 Hz of −82°, achieving capacitance of 175 µF and RC time constant of less than 200 µs, with predominant graphene edge charge storage rather than their basal plane charge storage.^131^ Following this pioneering work, several carbon‐based materials and polymers such as electrochemically reduced graphene oxide,^132^ carbon nanotube,^133^ carbon nanofiber aerogel,^134^ N‐doped graphene,^135^ and coordination and conducting polymers have been reported for high‐frequency supercapacitors, displaying high phase angles in the frequency range of tens to hundreds Hz.^136^

High electric and ionic conductive electrodes are essentially required for high‐frequency supercapacitors. Most of metal oxides and hydroxides are wide‐bandgap materials with low conductivity, leading to high series resistances when operating as supercapacitor electrodes. Thus, engineering routes of their charge storage materials or their composites with highly conductive matrixes to reduce resistivity are expected to provide better electrochemical response at high frequency. Although metal oxide and hydroxide–based electrodes are still scarcely studied for high‐frequency supercapacitors, some results have been reported in the literature showing adequate frequency response. For example, symmetric sandwich micro‐supercapacitors based on composites of V_2_O_3_/VO_2_–V_2_O_5_ core–shell nanostructures grown on graphene conductive framework displayed phase angles of ≈−80° and −50° at frequencies of 10 and 100 Hz, respectively.^137^ Symmetric planar supercapacitors based on reduced graphene oxide–MnO_2_–Ag nanowire hybrid film showed phase angles of ≈−90° over the frequency range of 10–100 Hz.^138^ Graphene quantum dot||MnO_2_ planar asymmetric micro‐supercapacitors showed phase angles of about −65° for frequencies up to 1 kHz.^139^ Although MnO_2_ single electrode studies were not reported in this work, the fast double‐layer charging at the graphene quantum dot negative electrode would enable the high‐frequency response of the whole asymmetric cell.

### 4.3 Flexible Supercapacitors

Currently, flexible and wearable electronic devices are being extensively studied as new generation devices in different applications. Flexible devices such as roll‐up displays, flexible sensors, transistors, optoelectronics, and antennas would not only increase their resistance against deformations but also extend the ways they can be integrated into different systems and upgrade our interaction on these devices. Currently used rigid supercapacitor cells such as cylindrical, coin, prismatic, and pouch cells are not easy to integrate in flexible devices. Thus, flexible supercapacitors are crucial to power up those systems.^140^ Assembling of flexible supercapacitors generally requires development of flexible components, including flexible electrodes and flexible electrolytes. Quasi‐solid‐state gel electrolytes are being widely used, thanks to their flexibility and reduced liquid leakage. Therefore, the development of flexible electrode has become the main spotlight. Different types of flexibility such as bending, twisting, stretching, and compressing have been studied. While simple bendable and twistable flexible supercapacitors are commonly achieved, stretchability and compressibility properties, which are important mechanical response for wearable devices, still face many challenges. Metal oxides and hydroxides are fractal materials, which can only sustain very small deformations without breaking. Thus, electrode engineering routes have been proposed, such as coating of those materials on flexible substrates or forming composites with other flexible materials. Note that most of the engineering approaches proposed for flexible high energy density supercapacitors can also be exploited for micro‐supercapacitors. The essential compatibility of micro‐supercapacitors with fiber‐based microelectronic or wearable devices opened a designing route for fiber‐based flexible micro‐supercapacitors. The ultimate goal is to achieve flexible electrodes and devices exhibiting high charge storage performance under different deformations.

**Figure 16:**
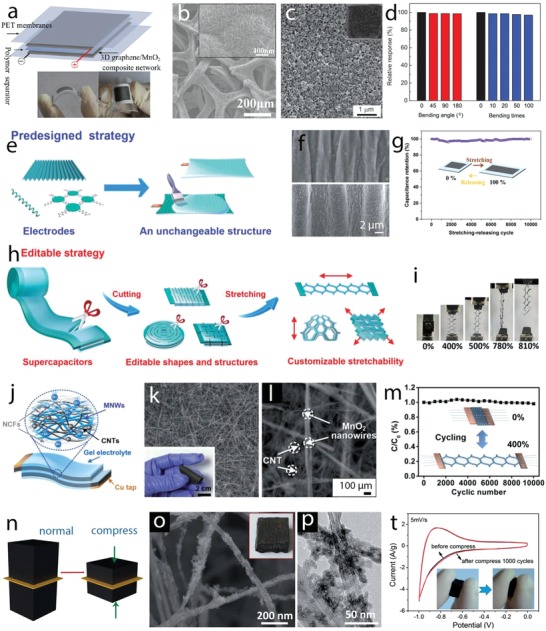
Flexible supercapacitors/electrodes: a,b) bendable symmetric MnO_2_–graphene||MnO_2_–graphene; c,d) bendable asymmetric carbon nanotube–ERGO||MnO_2_–ERGO; e–g) stretchable asymmetric Fe_2_O_3_–CNT||MnO_2_–CNT supercapacitors based on predesigned route^148^; h–m) stretchable symmetric MnO_2_–CNT||MnO_2_–CNT supercapacitors based on editable route; and compressible Fe_2_O_3_–CNT supercapacitor electrodes. a,e,h,j,n) Schematic illustration of supercapacitor cells and the formation of electrodes; b,c,g,k,l,o) FEG‐SEM images of the supercapacitor electrodes; d) capacity retention of the flexible supercapacitor after bending at different angles and repetitions; g) capacity retention at 1 A g^−1^ during 10 000 stretching–releasing cycles; i,m) the honeycomb‐shaped supercapacitors under stretching at different strains and the capacity retention at 1.6 mA cm^−2^ during 10 000 stretching/releasing cycles; m,t) cyclic voltammograms before and after compressing the electrode for 1000 cycles; p) TEM image of Fe_2_O_3_–CNT. a,b) Reproduced with permission.^143^ Copyright 2013, American Chemical Society. c,d) Reproduced with permission.^144^ Copyright 2014, John Wiley and Sons. e,h–m) Reproduced with permission.^149^ Copyright 2017, John Wiley and Sons. o,p,t) Reproduced with permission.[[qv: 151b]] Copyright 2015, Royal Society of Chemistry.

Most of flexible substrates such as stainless steel, Cu, Al, and carbon derivatives can only support bending and twisting, rendering this flexibility in many metal oxide and hydroxide–based flexible supercapacitors. Using preformed flexible substrates, the active metal oxide and hydroxide layers can only be integrated by the deposition route and the adhesion of active materials on their flexible substrates, without and with deformations, is very important to maintain long‐term functioning. For example, MnO_2_ and Fe_2_O_3_ films were grown on flexible stainless steel sheet and assembled into Fe_2_O_3_||MnO_2_ supercapacitors. These cells displayed good mechanical flexibility, operated in a wide working voltage of 2 V, and delivered energy density of 41.8 Wh kg^−1^ at power density of 1276 W kg^−1^.^141^ Compositing with carbon‐based materials enables the co‐growth of active materials and carbon into self‐supported flexible composite electrodes. The carbon networks enable the flexibility and conductivity of the electrodes and the redox‐active metal oxides and hydroxides enhance their charge storagecapacity. For example, MnO_2_–reduced graphene oxide composite bendable paper was prepared by vacuum filtration of graphene oxide solution containing Mn precursor followed by chemical reduction process.^142^ The asymmetric reduced graphene oxide paper||MnO_2_–reduced graphene oxide paper cells displayed bendability and delivered energy densities of 35.1 and 11.5 µWh cm^−2^ at power densities of 37.5 µW cm^−2^ and 3.8 mW cm^−2^, respectively. 3D porous flexible substrates such as foam‐like substrates (graphene foams), textiles (carbon cloths), and carbon aerogels have also been exploited. Advantages over flexible planar current collectors include increased charge storage capability and macroporous architectures that enhance the dispersibility of the redox materials increasing their surface active area and diffusibility of electrolyte ions. Moreover, 3D flexible substrates can sustain a certain compression, favoring the assembly of compressible supercapacitors. For example, MnO_2_ was electrodeposited on 3D graphene foam for assembling flexible MnO_2_–graphene foam||MnO_2_–graphene foam supercapacitors (**Figure** **16**a,b), which showed good bendability without loss of capacitance and good charge storage performance.^143^ Asymmetric carbon nantube (CNT)–electrochemical reduced graphene oxide foam (ERGOF)||MnO_2_–ERGOF cells displayed stable performance under different bending angles and times (Figure 16c,d).^144^ NiCo_2_O_4_ was grown on carbon cloth by a hydrothermal method and used as a positive electrode in flexible graphene paper||NiCo_2_O_4_–carbon cloth supercapacitors operating in PVA–LiOH gel electrolyte.^145^ This cell worked and was stable, with 96.8% capacitance retention after 5000 cycles, under mechanical twisted and bent conditions. Moreover, it delivered energy densities of 60.9 and 37.56 Wh kg^−1^ at power densities of 568.2 W kg^−1^ and 11.36 kW kg^−1^ within the working voltage of 1.8 V.

Stretchable and compressible supercapacitors require more demanding architecture designs than bendable and twistable supercapacitors. Stretchable designs by using stretchable substrates such as polymers in combination with predesigned stretchability electrode materials such as wave‐like architectures or bridge–island architectures (designed for micro‐supercapacitors) and self‐supported electrodes with similar architectures and using stretchable textile are being carried out (Figure 16e).^146^ Stretchable carbon electrodes have been developed, including graphene papers and single‐walled carbon nanotube films, that would promote the fabrication of the stretchable metal oxide/hydroxide–carbon composite electrodes for high energy density flexible supercapacitors.[[qv: 146b,147]] For example, stretchable Fe_2_O_3_–carbon nanotube||MnO_2_–carbon nanotube supercapacitors were reported.^148^ Fe_2_O_3_–carbon nanotube was prepared by chemical vapor deposition in the presence of Fe precursor and MnO_2_–carbon nanotube was prepared by redox deposition of MnO_2_ on etched Fe_2_O_3_–carbon nanotube (Figure 16f, bottom). These two self‐supported films were attached on prestretch polydimethylsiloxane (PDMS) and assembled with Na_2_SO_4_–PVA gel electrolyte, which was followed by release of the prestretch to form a wave‐like architecture and to enable stretchability (Figure 16f, top: MnO_2_–carbon nanotube, bottom: Fe_2_O_3_–carbon nanotube). These cells displayed excellent stretchability under tensile strains up to 100%, operated stably in the working voltage of 2 V (Figure 16g), and delivered energy density of 45.8 Wh kg^−1^ at power density of 406.6 W kg^−1^. Although predesigned strategies have enabled the development of stretchable supercapacitors, to enable compatibility with different devices, adaptable stretchability in different patterns is also an expected property. Moreover, mechanical mismatches between substrates, electrode materials, and gel electrolytes can lead to failure of supercapacitors assembled by predesigned approaches. Recently, stretchable supercapacitors with adaptability by cutting routes into different stretchable patterns were developed (Figure 16h).^149^ This supercapacitor was assembled in a symmetric design based on two MnO_2_ nanowire–carbon nanotube composite electrodes, which were separated and sealed by nanocellulose fiber films in LiCl–PVA electrolyte, forming a paper‐like cuttable supercapacitor (Figure 16j–l). By cutting route, different stretchable structures can be attained such as honeycomb‐like, pyramid pop‐up, and living‐hinge structures, displaying excellent stretchability and charge storage ability. For example, honeycomb‐like structures can be stretched up to 400% strain deformation and delivered stable charge storage capacity of 227.2 mF cm^−2^ during 10 000 stretching–releasing cycles (Figure 16i,m).

Stretchability is an extremely important property for wearable supercapacitors. Thus, textile‐based stretchable substrates and fiber/yarn‐based supercapacitors, which can be then woven or knitted into textiles, are under considerable studied and will be discussed at the end of this section together with flexible micro‐supercapacitors.

Compressible designs could be achieved by two different routes by employing compressible electrolytes or compressible substrates.^150^ Most of gel electrolytes currently used are compressible, favoring the assembly of compressible supercapacitors. Moreover, this route enables the use of noncompressible electrodes for compressible supercapacitors. Although the compressibility of different gel electrolytes is varied and can be optimized, a thin‐layer electrolyte is required to reduce their resistance; thus, compressibility is limited to millimeter scale. This limitation can be overcome by utilizing compressible substrates, which can display a compressible capability up to centimeter scale (Figure 16n). For example, composites of MnO_2_–carbon aerogels and α‐Fe_2_O_3_–carbon sponges displayed stable compressibility under 50% strain (Figure 16n,o,p,t).^151^ Electrode architecture designs could also form compressible electrodes, although this is rarely reported. MnO_2_–ultralong Ni nanowire grown on Ti foils displayed compressibility of 20% of their initial thickness, enabling an assembly of compressible supercapacitors.^84^ The compressible cells assembled with MnO_2_–ultralong Ni nanowire as positive electrode and polypyrrole–ultralong Ni nanowire as negative electrodes could be compressed about half of their thickness.

Flexible micro‐supercapacitors have been proposed to integrate in flexible microelectronic devices. Planar micro‐supercapacitors are typically fabricated on flexible substrates such as plastic and paper to enable their flexibility.^152^ Moreover, different electrode architecture engineering approaches have been employed to enhance their charge storage performance. For example, planar symmetric Au/MnO_2_/Au flexible micro‐supercapacitors, fabricated on PET substrates, showed good bendability and delivered areal capacitance of 11.9 mF cm^−2^ at 0.05 mA cm^−2^.[[qv: 152a]] Stretchable planar micro‐supercapacitors were also designed on stretchable polymer substrates.^153^ The stretchable substrate was engineered into two different regions, in which stiff island arrays (PDMS) distributed over a soft polymer (mixture of Ecoflex and PDMS) as base. Micro‐supercapacitors were designed in planar configuration consisting of Mn_3_O_4_–multiwall carbon nanotube composite microelectrodes, which were fabricated on the top of stiff islands and connected by embedded liquid metal interconnections. This design enabled stable operation of the micro‐supercapacitor arrays under stretching deformation with uniaxial strain up to 40%, in which the deformation occurs mainly on the soft polymer substrate.

**Figure 17:**
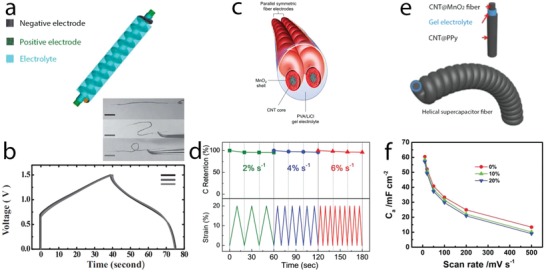
Wire/fiber/yarn‐based micro‐supercapacitors in twisting, paralleling, and coaxial designs of wire/fiber/yarn electrodes. a) Schematic representation of twisted asymmetric pen ink||Ni–Co hydroxide supercapacitor and b) charge–discharge plots at 0.1 mA (bottom) under different bending of ordered mesoporous carbon–Ni fiber||Ni(OH)_2_ nanowire–Ni fiber (top). Schematic representations and electrochemical response under different flexibility tests of c,d) paralleled symmetric MnO_2_–CNT||MnO_2_–CNT coiled yarn supercapacitor and e,f) coaxial asymmetric polypyrrole–carbon nanotube||MnO_2_–carbon nanotube fiber supercapacitor. d) Capacitance retention under different bending strain rates; f) capacitance values under different stretching strains and scan rates. a) Reproduced with permission.[[qv: 154b]] Copyright 2017, American Chemical Society. b) Reproduced with permission.[[qv: 154a]] Copyright 2014, John Wiley and Sons. c–f) Reproduced with permission.[[qv: 155b,156b]] Copyright 2016, John Wiley and Sons.

Together with the development of wearable devices, the development of flexible fiber‐based microelectronics has led to the growth of wire/fiber/yarn‐based micro‐supercapacitors to integrate with their devices for power supplying. Their fiber‐based supercapacitors have been assembled by three different architectures, including twisting,^154^ paralleling of two fiber electrodes,^155^ or designing them in coaxial structures (**Figure** **17**a,c,e).^156^ Currently, metal and carbon fibers/yarns are essentially employed as supports for fabrication of metal oxide and hydroxide–based fiber electrodes to enable flexibility.[[qv: 154a,157]] While metals can barely act as conductive supports, carbon fibers have been exploited as both conductive support and charge storage material via activation or oxidation of carbon materials to increase their double‐layer or pseudocapacitive charging.^158^ Note that for fiber‐shaped supercapacitors, capacity normalized by unit of length or area is the most important capacity metric among others. For example, twisted asymmetric fiber‐shaped and ordered mesoporous carbon–Ni fiber||Ni(OH)_2_ nanowire–Ni fiber supercapacitors displayed capacity of 6.67 mF cm^−1^ (35.67 mF cm^−2^) at 0.1 mA, energy density of 0.01 mWh cm^−2^, and operated normally at different bending states (Figure 17b).[[qv: 154a]] Carbon fibers were also coated with a metallic conducting layer such as Ni to increase their support conductivity.[[qv: 154b]] Twisted asymmetric pen ink||Ni–Co hydroxide supercapacitors based on Ni‐coated carbon fibers (Figure 17a) showed good charge storage performance with stretchable deformation. Carbon nanotubes were oxidized to increase their charge storage capacity, followed by a deposition of MnO_2_ to form MnO_2_–oxidized carbon nanotube fiber electrodes.^158^ The paralleled symmetric fiber cells constructed by the two paralleled fiber electrodes were assembled by a prestrained route to enable stretchability. The synergistic contribution to the charge storage of oxidized carbon nanotube and MnO_2_ resulted in high‐capacity stretchable fiber supercapacitors, displaying capacitance values of ≈409.4 F cm^−3^ (or 133 mF cm^−2^) at 0.75 A cm^−3^. The paralleled symmetric MnO_2_–CNT||MnO_2_–CNT coiled yarn supercapacitor was assembled with MnO_2_‐coated CNT coiled yarn (Figure 17c).[[qv: 155b]] This microcell presented a static capacitance value of 2.72 mF cm^−1^, which retained 84% on stretching with 37.5% strain, and the static capacitance retained 96.3% under dynamic test at 20% strain deformation and strain rate of 6% s^−1^ (Figure 17d). Coaxial asymmetric polypyrrole–carbon nanotube||MnO_2_–carbon nanotube fiber supercapacitors were assembled with MnO_2_–carbon nanotube as core, which was coated with a KOH–PVA gel electrolyte and the polypyrrole–carbon nanotube composite as an outer layer (Figure 17e).[[qv: 156b]] Moreover, this coaxial fiber supercapacitor was further twisted into a helical structure to enable stretchability. This cell displayed quite good reversible charge storage under stretching up to 20% (Figure 17f) and delivered energy density of 18.88 µWh cm^−2^.

### 4.4 Smart Supercapacitors

#### 4.4.1 Multipurpose Supercapacitors

Previous sections discussed supercapacitors as power devices for high energy density storage, microelectronics, and applications requiring flexibility. For electronic and portable devices, the integration area and volume of different electronic components are the key for miniaturization; thus, electronic components, displaying multiple functionalities or different properties, can advance miniaturized devices. Currently, multipurpose electronic components such as light‐emitting transistors, light‐emitting photodetector diodes, and electrical–optical transistors are widely used. Therefore, the development of multipurpose supercapacitors can further advance the next generations of miniaturized devices. Note that this section discusses supercapacitors displaying multipurpose functionalities; the integration of supercapacitors with other devices is also under development to achieve multipurpose systems, but this topic is out of scope of this section.^159^

**Figure 18:**
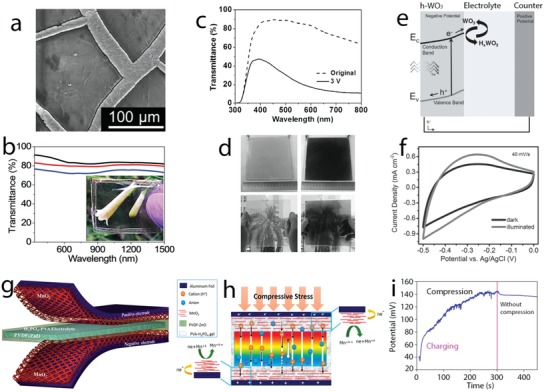
Multipurpose supercapacitors. a,b) FEG‐SEM image and transmittance spectra of MnO_2_–Au network (black curve: Au network electrode; red curve: MnO_2_–Au network electrode; blue curve: assembled symmetric MnO_2_–Au network supercapacitor); inset: photo of MnO_2_–Au network on a separator. c,d) Transmittance spectra of symmetric WO_3_ supercapacitor upon assembling and charging and photos at bleached and colored states. e,f) Schematic illustration of the enhanced charge storage upon illuminating with solar light and cyclic voltammograms at 40 mV s^−1^ under dark and illumination. g) Schematic representations of the self‐charging supercapacitor and h) the charge storage processes and i) self‐charging plot under piezoelectric field on compressing. a,b) Reproduced with permission.[[qv: 161a]] Copyright 2017, John Wiley and Sons. c,d) Reproduced with permission.^165^ Copyright 2014, John Wiley and Sons. e,f) Reproduced with permission.[[qv: 169a]] Copyright 2016, John Wiley and Sons. g–i) Reproduced with permission.^170^ Copyright 2015, American Chemical Society.

Supercapacitors displaying properties such as optical transparent, electrochromic, sensing, lighting, and energy harvesting have been reported. Among those, transparent and electrochromic supercapacitors are currently successful multipurpose devices. Transparent supercapacitors are being developed via two main routes, including i) assembling on transparent supports^160^ and ii) as paper‐like freestanding supercapacitors.^161^ For example, transparent supercapacitors based on symmetric MnO_2_–Au nanonetworks on a separator were reported.[[qv: 161a]] The MnO_2_–Au nanonetwork architecture was prepared by evaporation of Au on a cracked separator followed by electrodeposition of MnO_2_ and enabled transparency with transmittance of 72% (λ = 550 nm) and charge storage capacity of 3 mF cm^−2^ at 5 µA cm^−2^ (**Figure** **18**a,b). Symmetric paper‐like self‐standing transparent supercapacitors were assembled using transparent α‐MoO_3_ papers, which were prepared by vacuum filtration of ultralong α‐MoO_3_ (200 µm in length) and displayed transmittance of ≈90% in the visible region.[[qv: 161b]] This supercapacitor showed capacitance values of 257.6 F g^−1^ at 5 mV s^−1^ and 94.6 F g^−1^ at 200 mV s^−1^ and 96.5% capacitance retention after 20 000 cycles.

Some redox‐active materials display color changes during charge and discharge processes, which is also known as electrochromic property. This property enables supercapacitor devices operating as both power supply devices and electrochromic devices. Moreover, color changes due to electrochromic effect depend upon charge states of supercapacitors, being a smart energy level indicator of supercapacitors.^162^ The bifunctional devices allow efficient use of energy, in which the devices can store charge during the electrochromic color changes and can function as power source during color bleaching processes. These advantages lead to the growing research in electrochromic supercapacitors. Several metal oxides/hydroxides and their composites have been recently reported for aqueous electrochromic supercapacitors such as NiO,^163^ W–Mo oxides,^164^ WO_3_,^165^ Ni(OH)_2_–MoO_3_,^166^ and Co_1−_ *_x_*Ni*_x_*(OH)_2_–rGO.^167^ For example, symmetric electrochromic supercapacitors consisting of thin‐film electrodes of WO_3_ nanoparticles delivered areal capacitance of 12.8 mF cm^−2^ at 0.4 mA cm^−2^ and showed optical transmittance of 78.8% in a bleached state and 15.1% in a colored state at a wavelength of 633 nm (Figure 18c,d).^165^ Asymmetric carbon nanotube||Ni(OH)_2_–MoO_3_ electrochromic supercapacitors showed optical transmittance of 60% in the bleached state and 16% in the colored state at a wavelength of 500 nm.^166^ The charge storage and electrochromic processes were assigned to proton deintercalation/intercalation in layered WO_3_ or Ni(OH)_2_.

Photoelectrochemical cells are among possible ways to convert solar energy into electrical energy. The presence of a space charge region upon photoexcitation immediately results in reduction reactions and oxidation reactions with generated electrons and holes, respectively. Thus, storing charge via photoelectrochemical processes has been a difficult task. Nevertheless, under certain conditions, electron–hole pairs can be separated and electrons could be stored, opening a concept of solar energy harvesting supercapacitors. For example, pseudocapacitive materials such as layered WO_3_ and MoO_3_ showed photocharging and storage ability, where the photoexcited electrons were stored via intercalation reactions with cations and photoexcited holes oxidize absorbed water to generate protons, thus balancing the total charge and suppressing electron–hole pair recombination to allow charge storing.^168^ In another approach, photoexcited electrons can be trapped by electrolyte cations and stored together with typical charge storage processes of supercapacitor electrodes, thanks to the applied voltage bias when charging supercapacitors.^169^ For example, symmetric Co(OH)_2_ supercapacitors showed enhanced areal capacity under blue light illumination, achieving capacity of 4.7 mF cm^−2^ at 0.1 mA cm^−2^, which is 2.4 times higher than a capacity obtained under dark conditions.[[qv: 169b]] Hexagonal WO_3_ showed ≈17% enhancement of charge storage capacity under solar light illumination compared to the value obtained under dark because its bandgapenergy is close to visible light energy (Figure 18e,f).[[qv: 169a]] The capacity of WO_3_‐based supercapacitors varied under light illumination with different wavelength and intensity, which corresponds to changes of discharge time and voltage drop. Thus, this response difference between dark and illumination environments was employed to detect light, being an indicator for the bifunctional photodetecting supercapacitors.

By using piezoelectric materials as separator, when assembling supercapacitors, self‐chargeable supercapacitors have been proposed.^170^ The self‐charging supercapacitor (this concept represents supercapacitors that can be charged without using external power sources) was designed in symmetric configuration, using MnO_2_ nanowire electrodes and a piezoelectric polyvinylidene difluoride–ZnO separator, which were immersed in PVA–H_3_PO_4_ gel electrolyte (Figure 18g). Under vertical compression to the large dimension size of the supercapacitor, the piezoelectric separator generated a voltage causing positive and negative polarizations on opposite sides. This polarization drove electrolyte ions to positive and negative electrodes and created in‐equilibrium electrode–electrolyte interface, which led to proton intercalation/deintercalation redox reactions at negative/positive MnO_2_ electrodes (Figure 18h). These processes autonomously charged the supercapacitor, without need of external power sources. Under compression for 300 s (hand pressing), the voltage of the supercapacitor increased from 35 to 145 mV (Figure 18i). Its self‐charging capability was enhanced by increasing compression force, thanks to an enhanced piezoelectric potential polarization. Moreover, it also showed stable capacity under self‐charging and discharging at 10 µA.

#### 4.4.2 Restorable and Degradable Supercapacitors

**Figure 19:**
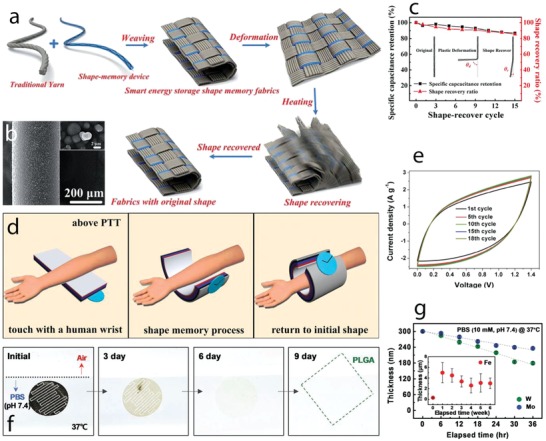
Restorable and biodegradable supercapacitors. a) Schematic representation of a shape‐memory textile knitted with shape‐memory fiber supercapacitors (SMFSCs); b) FEG‐SEM image of NiTi wire coated with MnO_2_ and polypyrrole layers; c) capacitance retention and shape recovery ratio of SMFSCs at different shape recovering cycles. d) Schematic representation of shape recovering of a shape‐memory watchband after touching with a wrist; e) cyclic voltammograms of the shape‐memory supercapacitor during dynamical shape change and restoration at 100 mV s^−1^. f) Optical photos of Mo electrode dissolution when immersed in PBS solution at 37 °C; g) thickness changes of W, Fe, and Mo thin films (thickness of 300 nm) at different immersed times in PBS. a–c) Reproduced with permission.^171^ Copyright 2015, Royal Society of Chemistry. d,e) Reproduced with permission.^172^ Copyright 2016, John Wiley and Sons. f,g) Reproduced with permission.^178^ Copyright 2017, John Wiley and Sons.

On practical use, supercapacitor devices can undergo different intentional and accidental deformations. Flexible supercapacitors using polymer substrates may fail due to irreversible plastic deformation. Thus, devices with restorable capabilities, i.e., able to recover their initial shape upon mechanical deformation, under external triggers are highly desirable. This sound concept has recently been achieved by employing shape‐memory alloys as current collectors.^171^ These alloys provide two important properties for restorable supercapacitors: i) the shape‐memory effect and ii) superelasticity, which can be restored to their initial shape under heating, thanks to the temperature‐induced reversible martensite–austenite phase change. Pioneering work demonstrated the use of NiTi shape‐memory alloy wires as current collectors, which were coated with pseudocapacitive layers consisting of MnO_2_ in the inner layers and polypyrrole in the outer layers (**Figure** **19**b), and assembled in twisted symmetric wire‐shape supercapacitors.^171^ This device showed stable performance under bending, twisting, knotting, release, and repeated bending tests. Interestingly, by heating the supercapacitor to temperatures above the austenite phase transformation, the deformed supercapacitor was restored to its initial shape, without significant capacity loss, even under repeating deformation–restoration tests (Figure 19c). Knitting the shape‐memory supercapacitors into textile resulted in a restorable textile (Figure 19a). The heat‐induced restoring temperature was about 35 °C. This value is close to human body temperature; thus, the restorable textile can be used in smart clothes for autonomous heat dissipation (by shape changes) when the body temperature increases. Using a similar concept and making use of shape‐memory alloys, a smart watchband supercapacitor with automatic wrap ability in contact with skin has been reported (Figure 19d).^172^ The asymmetric supercapacitor consisted of reduced graphene oxide on a shape‐memory TiNi foil as negative electrode and MnO_2_ onto a Ni film as positive electrode, showing stable performance under static bending at different angles and dynamic shape‐memory restoration bending (Figure 19e). The shape‐memory alloy showed a phase transformation temperature at 15 °C, leading to a preformed round‐shape watchband supercapacitor, which under bending states at low temperature (0 °C) could be automatically restored to its initial shape and wrapped to our wrist upon touching (Figure 19d).

Deformations accumulated on supercapacitors during operation can generate mechanical failures, stopping their operation. In these cases, the shape restoration is not appropriate to repair failures. Thus, the capability to heal mechanical failures induced by the deformations would provide another innovative way to recover supercapacitor functionality. Thanks to healable materials, a self‐healing symmetric supercapacitor has been achieved by using healable materials as substrates to disperse carbon nanotubes working as double‐layer charging materials and current collectors.^173^ Under mechanical breaking, the supercapacitor could be healed by joining the cut sections under small pressure; the healable substrate could drag the displaced carbon nanotube layer together, thereby recovering conductivity and charge storage capability, with ≈82% of the initial capacitance restored after five cutting–healing cycles. Wire‐shape healable supercapacitors,^174^ using magnetic nanoparticles as internal trigger to join healable materials, were recently reported.^175^ Although metal oxide and hydroxide–based healable supercapacitors have not been reported yet, to the best of authors' knowledge, from the above conceptual work, the coating of these active materials on conducting percolation networks such as carbon materials or metals followed by their dispersion on or wrapping with healable layers would enable self‐healing supercapacitor forms with enhanced capacity. Recently, a detailed review on healable materials was published,^176^ which would provide more insights into healing materials and mechanisms for further studies in healable energy storage devices.

Integration of supercapacitors with biocompatible electronics to power up bioresorbable implanted devices and development of eco/bioresorbable supercapacitors (transient power devices) are presently under consideration. Bioresorbable supercapacitors can slowly degrade and dissolve in physiological media after working for a specified period, eliminating concerns about toxicological issues and additional surgeries for removal. Recently, bioresorbable supercapacitors were assembled using different types of biomaterials such as charcoal electrodes, egg white binders, polyelectrolyte drink, cheese segregation layer, and seaweed separator, forming an eatable device.^177^ However, the concept of eatable devices does not meet the principal requirement of bioresorbable devices, that is, to degrade after stable operation for a certain period. More recently, a biodegradable planar micro‐supercapacitor was reported, being composed of transition metals (W, Fe, or Mo) as electroactive materials and current collectors and biodegradable NaCl–agarose biopoly‐mer gel electrolytes. These materials were assembled together with a biodegradable polymer poly(lactic‐*co*‐glycolic acid) film and encapsulated with biodegradable hydrophobic polyanhydride.^178^ Metal oxide layers formed by metal corrosion and oxidation during charging/discharging process provided redox charge storage with enhanced capacity. This flexible device delivered a capacitance of 0.33 mF cm^−2^ at 0.15 mA cm^−2^, which is comparable to other micro‐supercapacitors and displayed stable performance, facilitating integration in different systems. Metal electrodes (Mo and W) dissolved in phosphate‐buffered saline (PBS) at body temperature as evidenced in the dissolution rate (by thickness change) plot (Figure 19g). A Mo micro‐supercapacitor, without encapsulation, could operate in PBS at physiological temperature for 6 h, being completely dissolved after 9 days (only the biodegradable substrate was left because of its longer degradation time) (Figure 19f). Encapsulation with hydrophobic polyanhydride expanded the working time up to 2 weeks.

Several smart supercapacitors tailored for different applications have been reported. Although the concepts are all very exciting, some of those are not of practical use yet. For example, the self‐charging supercapacitor can only charge from 35 to 145 mV, which is far from application. Thus, supercapacitor components—including electrode materials, electrolytes, and separators—should be investigated further to enable the development of smart device assemblies. An interesting research path is the development of biocompatible supercapacitors, active materials, current collectors, and electrolytes to power up implantable systems. This is still at the concept level, but opens new routes to design smart devices tailored to serve a novel array of applications.

## 5 Concluding Remarks

This review comprehensively highlighted and discussed the most important advances from the authors' viewpoints in metal oxide and hydroxide–based aqueous supercapacitors over the recent years, spanning from fundamental charge storage mechanisms, electrode materials engineering and functionalization, to tailored supercapacitor devices for multipurpose applications. Significant achievements have been attained during that last few years, including deeper understanding of the mechanisms governing charge storage, different electrode engineering routes to enhance electrochemical performance, and the design of flexible and/or smart devices. Despite considerable progress, there are still many research gaps to be filled and this paves the way for new exciting research streams in the near future.

Concerning fundamental charge storage mechanisms, often contradictory results have been reported, probably because studies are based on a limited number of electrochemical studies and ex situ characterization. Therefore, collaborative studies based on different in situ/operando techniques are necessary to achieve deeper comprehension on the charge storage mechanisms of different electrode materials.

Although some advances in electrode engineering have been made, energy density values are still modest. Often the reported values are generally based on laboratory‐based studies, using electrodes with low mass loadings. Normally these electrodes are assembled with few milligrams of active material, while commercial carbon‐based supercapacitors are typically assembled with mass loadings of one order of magnitude above. It has been shown that metal valences during the charge–discharge process are not fully oxidized or reduced, or only a part of the active materials is participating in the charge–discharge processes. Further electrode materials engineering routes that explore the full potential of charge storage by valence changes of metal ions are absolutely crucial to fully explore the potential of metal oxides and hydroxides. Moreover, important advances are still required to design electrodes with optimal properties (surface area, phase composition, doping/vacancies, electron conductivity, and architecture) for enhancing charge storage performance and cycling ability.

Smart supercapacitors, tailored for different applications, can exhibit remarkable capabilities such as restorability and degradability. Although some initial results have been reported, this strategy is still at early research, but certainly future studies on this stream will pave the way toward many innovative results and hi‐tech applications.

In conclusion, presently, aqueous supercapacitors based on metal compounds are a very dynamic research stream, in which understanding charge storage mechanisms, optimized electrode engineering, and tailored device assemblies are the main challenges to develop high‐performance devices and to meet the specific needs of the application.

## Conflict of Interest

The authors declare no conflict of interest.
